# A theory of memory for binary sequences: Evidence for a mental compression algorithm in humans

**DOI:** 10.1371/journal.pcbi.1008598

**Published:** 2021-01-19

**Authors:** Samuel Planton, Timo van Kerkoerle, Leïla Abbih, Maxime Maheu, Florent Meyniel, Mariano Sigman, Liping Wang, Santiago Figueira, Sergio Romano, Stanislas Dehaene

**Affiliations:** 1 Cognitive Neuroimaging Unit, CEA, INSERM, Université Paris-Sud, Université Paris-Saclay, NeuroSpin center, Gif/Yvette, France; 2 Université de Paris, Paris, France; 3 Laboratorio de Neurociencia, Universidad Torcuato Di Tella, Buenos Aires, Argentina; 4 CONICET (Consejo Nacional de Investigaciones Científicas y Tecnicas), Buenos Aires, Argentina; 5 Facultad de Lenguas y Educacion, Universidad Nebrija, Madrid, Spain; 6 Institute of Neuroscience, Key Laboratory of Primate Neurobiology, CAS Center for Excellence in Brain Science and Intelligence Technology, Chinese Academy of Sciences, Shanghai, China; 7 Universidad de Buenos Aires. Facultad de Ciencias Exactas y Naturales, Departamento de Computacion, Buenos Aires, Argentina; 8 Collège de France, Paris, France; McGill University, CANADA

## Abstract

Working memory capacity can be improved by recoding the memorized information in a condensed form. Here, we tested the theory that human adults encode binary sequences of stimuli in memory using an abstract internal language and a recursive compression algorithm. The theory predicts that the psychological complexity of a given sequence should be proportional to the length of its shortest description in the proposed language, which can capture any nested pattern of repetitions and alternations using a limited number of instructions. Five experiments examine the capacity of the theory to predict human adults’ memory for a variety of auditory and visual sequences. We probed memory using a sequence violation paradigm in which participants attempted to detect occasional violations in an otherwise fixed sequence. Both subjective complexity ratings and objective violation detection performance were well predicted by our theoretical measure of complexity, which simply reflects a weighted sum of the number of elementary instructions and digits in the shortest formula that captures the sequence in our language. While a simpler transition probability model, when tested as a single predictor in the statistical analyses, accounted for significant variance in the data, the goodness-of-fit with the data significantly improved when the language-based complexity measure was included in the statistical model, while the variance explained by the transition probability model largely decreased. Model comparison also showed that shortest description length in a recursive language provides a better fit than six alternative previously proposed models of sequence encoding. The data support the hypothesis that, beyond the extraction of statistical knowledge, human sequence coding relies on an internal compression using language-like nested structures.

## Author summary

Sequence processing, the ability to memorize and retrieve temporally ordered series of elements, is central to many human activities, especially language and music. Although statistical learning (the learning of the transitions between items) is a powerful way to detect and exploit regularities in sequences, humans also detect more abstract regularities that capture the multi-scale repetitions that occur, for instance, in many musical melodies. Here we test the hypothesis that humans memorize sequences using an additional and possibly uniquely human capacity to represent sequences as a nested hierarchy of smaller chunks embedded into bigger chunks, using language-like recursive structures. For simplicity, we apply this idea to the simplest possible music-like sequences, i.e. binary sequences made of two notes A and B. We first make our assumption more precise by proposing a recursive compression algorithm for such sequences, akin to a “language of thought” with a very small number of simple primitive operations (e.g. “for” loops). We then test whether our theory can predict the fluctuations in the human memory for various binary sequences Using a violation detection task, across many experiments with auditory and visual sequences of different lengths, we find that sequence complexity, defined as the shortest description length in the proposed formal language, correlates well with performance, even when statistical learning is taken into account, and performs better than other measures of sequence complexity proposed in the past. Our results therefore suggest that human individuals spontaneously use a recursive internal compression mechanism to process sequences.

## Introduction

Sequence processing, the ability to encode and represent in memory a temporally ordered series of discrete elements, plays a central role in numerous human activities, including language. In the 1950’s, Karl Lashley [1] and Noam Chomsky [2] famously argued that the sequential structures that humans produce and remember cannot be reduced to mere associations of consecutive items, as envisaged in the associative theories characteristic of the Skinnerian paradigm, but must be mentally represented as recursively nested structures. The syntax of language, for instance, involves a recursive grammar of potentially unlimited embeddings of phrases within phrases, and a similar argument has been made for a “musical grammar” [3]. Here, we formulate and test the theory that a similar code is needed to account for the much simpler case of binary sequences, i.e. sequences composed of two items A and B (e.g. high and low pitch tones, or red and green dots). We present experimental evidence that, even in this simple case, which can be considered as the simplest possible form of “music”, a similar postulation of nested structures is required in order to account for human memory performance.

Understanding how humans and other animals encode and represent temporal sequences has recently emerged as a crucial issue in the study of comparative cognition, as it allows a direct comparison between species and therefore a test of theories of human uniqueness [4,5]. Recursive phrase structures have been proposed to lie at the core of the human language faculty [6], and a competence for nested trees has been postulated to underlie several other human cognitive abilities such as mathematics or music [4,7–9]. According to a recent review [4], non-human animals may encode sequences using a variety of encoding schemes, including transition probabilities, ordinal regularities (what comes first, second, etc.), recurring chunks, and algebraic patterns [10–14]. However, several authors hypothesize that only humans have access to a language-like representation of nested trees [4,8], also being described as a “universal generative faculty” [9] or “language of thought” [15] capable of encoding arbitrarily nested rules.

Here we propose a principled language capable of encoding any arbitrary nesting of repetition and alternation structures, and we test the hypothesis that humans spontaneously encode sequences using the nested tree structures of this language. We do so using the simplest form of temporal sequences, namely binary sequences. Indeed, while the use of recursive chunking and embedding strategies is well accepted for richer sequences (e.g., language, music, or even memorizing a phone number [16]), it is not clear whether these mechanisms only become necessary at a certain level of complexity, or whether they lie at the core of human sequence processing and are therefore spontaneously employed even with the most basic forms of sequences. In addition to being the simplest possible such form, binary sequences also present several advantages. As opposed to more complex sequences, such as the ones of the natural language, which involve numerous factors that are difficult to control (prior knowledge, semantic content, word frequency, etc.), they allow to easily control the information content of the input. Furthermore, they are potentially accessible to a wide variety of populations beyond human adults, including infants and non-human primates. As such, they may provide an essential benchmark in research on the existence of a human-specific sequence processing ability. Finally, binary sequences are also widely used to study the cognitive processes and brain mechanisms involved in the perception of randomness and in statistical learning [17–22]. While minimal, they nevertheless preserve the possibility of forming structures at different hierarchical levels, from simple chunking to language-like rules, and thus of arbitrating between different models of sequence encoding.

### A short review of theories and experiments on sequence complexity

The concept of compression in working memory has a long history. Much research shows that human memory is not simply determined by the number of words, digits or locations that must be remembered, but also by their capacity to be “compressed” into a smaller number of known phrases, groups, or chunks [23–29]. The apparent discrepancies between the different limits of working memory capacity proposed in the past, e.g. 7±2 items [29] versus 4 items [25,30] can indeed be reconciled if one takes into account the possibility of constituting chunks rather than encoding a complete series of individual items [16,31]. The formation of chunks can be seen as a data compression process, and it was proposed that the complexity of a sequence can be defined as the size of its most compressed representation [16,32–34].

Experimentally, half a century of behavioral studies has shown that accuracy in sequence encoding and production tasks varies according to the compressibility of the sequence. Glanzer and Clark [35] already proposed to use the length of the most compact description of a sequence as a measure of its complexity. They found that the number of words that participants used to describe an array of eight binary items (colored symbols) was correlated with the accuracy in reproducing it. Such *mean verbalization length* (MVL) predicted behavior better than a simple count of the number of runs in the sequence (e.g. “AAABBBAA” has three runs), particularly for the “ABABABAB”, which could be simply described as “alternating”.

Generalizing upon this early work, one may propose that the complexity of a sequence relates to the length of its compressed form when it is recoded using an internal language. Consistent with such idea, Restle and Brown [36] showed that participants learned a series of 10 button presses, not as an associative chain of elements, but by encoding it as an abstract pattern, defined as the set of rules that were needed to generate it. The profile of errors suggested that participants represented the sequences as hierarchical trees of embedded rules (i.e. repetition, transposition, mirroring), equivalent to the tree structures found in language [37]. The psychological reality of this proposal was strengthened by showing that performance decreased precisely at the boundaries of higher hierarchical level groups of elements [36–38]. However, this approach was not developed into a full-blown universal language explaining how any sequence or pattern would be encoded.

A more formal approach for estimating the complexity of patterns, usually referred to as algorithmic complexity, program size complexity, or *Kolmogorov complexity* (KC), was proposed by Kolmogorov [39], Chaitin [40] and Solomonoff [41], within the framework of “algorithmic information theory”. These mathematicians defined the complexity of a sequence as the length of the shortest computer program capable of producing it. Strictly speaking, the algorithmic complexity is defined relative to a specific descriptive language (or programming language). When this language is Turing complete—which means that one can simulate any other Turing machine on it—we talk about universal or plain KC. Unfortunately, since it is impossible to determine whether any universal Turing machine will halt or not, KC is not computable. However, when the encoding language has reduced expressive power (i.e. when it is a *specific* machine rather than an *universal* machine), algorithmic complexity can be calculated and used as a subjective measure of complexity [42]. Recently, the group of Gauvrit, Delahaye, Zenil and Soler-Toscano proposed an approximation to KC using the “coding theorem”, which relates the algorithmic complexity of a sequence to the probability that a universal machine outputs that sequence [43–46]. They provided algorithmic complexity measures for a large set of short sequences. This proposal was presented as the best approximation of “an ultimate measure of randomness” and appeared to predict the biases observed when individuals are asked to either judge the randomness of patterns or to produce random patterns [44,45].

As an alternative to algorithmic complexity, Aksentijevic and Gibson [47] proposed another measure of sequence complexity, based on the notion of “change” (the inverse of invariance), which they called *change complexity*. They argued that humans attend to the structural information conveyed by the transition from one item to the next, rather than to the symbols themselves. Change complexity is thus computed by quantifying the average amount of change across all sub-sequences contained in a sequence. Aksentijevic and Gibson [47] further show that their measure has interesting properties such as a sensitivity to periodicity and symmetries, and that it performs better than previously proposed measures in predicting objective behavioral performance and subjective complexity of sequences.

As stated above, a proposal tightly related to KC is that human subjects compress sequences internally, not necessarily using a set of instructions of a Turing-complete language, but using a variety of computer-like primitives such as for-loops, while-loops, and other routines forming a specific internal “language of thought” [15], strong enough to describe any sequence, but not Turing complex and therefore weak enough to permit an explicit computation of complexity. Such a language would allow the combination of simple primitives into complex embedded patterns or recursive rules. Language of thought (LoT) models have been proposed very early on [34]. Simon & Kotovsky [48] used concepts such as “same”, “next” (on the alphabet), and the ability to cycle through a series, to build a formal representation of the human memory for sequences of letters (e.g. “cadaeafa…”). Similarly, Restle [37] used the operations “repeat”, “transposition” and “mirror image”. Similar languages, based on repetitions with variations, were also used to encode linear geometric figures and more elaborated 2D and 3D shapes [33,49]. More recently, similar proposals have been used with success to study different aspects of human learning, particularly concept learning [27,50–54]. Boolean complexity, i.e. the length of the shortest logical expression that captures the concept (a notion closely related to KC), was shown to capture human behavior in concept learning [27,55]. Going beyond the pre-specification of a specific language, the LoT approach has also been used to specify which grammar and which set of primitive operations best captures the behavior of human subjects [e.g. 56,57].

### The proposed language for binary sequences

The development of a LoT model for sequence representation involves the selection of a set of rules or operations whose combination allows the (lossless) recoding of any given sequence. We introduce here a formal language for sequence processing which is a variant of the *language of geometry* previously introduced by our team to model human performance in the domain of spatial working memory [58]. In this previous study, human participants were presented with a sequence of eight locations on a regular octagon. Using both behavioral and brain-imaging data, we showed the necessity and adequacy of a computer-like language consisting of geometrical primitives of rotation and symmetry plus the ability to repeat them with variations in starting point or symmetries [57–60]. This language was shown to predict which sequences appear as regular, and how educated adults, uneducated Amazon Indians and young children performed in an explicit sequence completion task [58] or in an implicit eye-tracking task [60]. Sequence complexity, defined as minimal description length, also predicted human brain activation in a broad cortical circuit including inferior frontal cortex just dorsal to Broca’s area [60].

Our language of geometry enables the generation of programs that can encode any sequence of spatial locations on an octagon. It uses primitive instructions (or rules) regarding the size and the direction of the next step (e.g. +1 = next element clockwise; +2 = second element clockwise), as well as the reflection over some axes (e.g. H = horizontal symmetry, picking the symmetrical location along a horizontal axis). Furthermore, these elements can be repeated, for instance +1^8 describes a full clockwise turn around the octagon (“^8” indicating a repetition of the instruction 8 times). Finally, those repetitions can be arbitrarily embedded (here denoted by brackets). For instance, the expression [[+2]^4]^2<+1> first draws a square, as determined by the subexpression [+2]^4, then a second one (denoted “[…]^2”) with an offset of +1 in the starting point (denoted by “<+1>”; see [58], for a full formal description).

**Fig 1:**
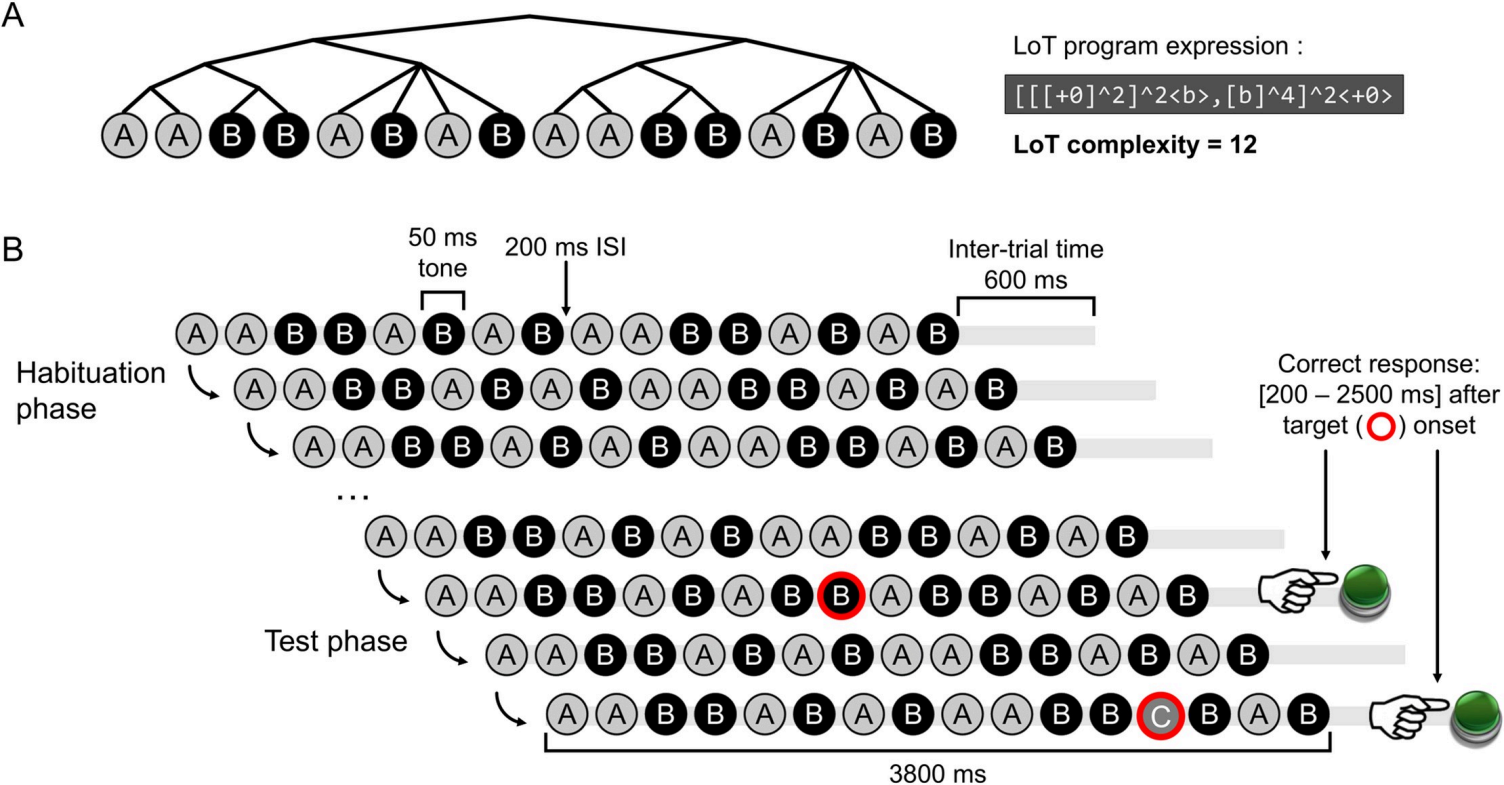
(A) Example of a 16-items long sequential pattern, with its shortest representation in the language of thought (i.e. LoT program expression) and the tree-structure derived from this expression (illustrating the hierarchical representation). The LoT complexity of this sequence is also indicated. (B) Experimental design of the violation detection task: a session with the sequence AABBABABAABBABAB is represented, with one example target deviant item (“A” replaced by “B”, at position 9) and one example target super-deviant item (“C” at position 13). Deviants could occur at positions 9, 11, 13 or 15.

In the present study, we test the highly constrained hypothesis that the same language, when reduced to only two locations, suffices to account for the human encoding of a completely different type of sequence, namely non-spatial (auditory and visual) binary sequences composed of only two arbitrary states A, B instead of the eight locations of the octagon. For such sequences, the language can be stripped of most of its primitives. We kept only the operations of staying (“+0”), moving to the other item (here denoted “b”, i.e. the alternation instruction, but equivalent to +4 or point symmetry in the original octagon-based language), and repetition (“^n”, where n is any number), possibly with a variation in the starting point (denoted by <x> where x is an elementary instruction, either +0 or b). As already mentioned, embedding of expressions is represented by brackets (“[….]”) and concatenation by commas (“,”). The language is thus able to encode any arbitrary repetition of instructions in a compressed manner. The sequence AAAA, for instance, would be denoted [+0]^4 (i.e. stay in the same state four times), the sequence ABAB would be denoted [+0]^4<b> (four repetitions, with an item change after each one; i.e., four alternations). The language is recursive and can produce nested descriptions; AABAAB can be described as “two repetitions of [two repetitions plus one change]” (see examples in Fig 1A). Because of recursion, even long sequences can be encoded compactly in an easy-to-remember form; ABABABBBBBBBABABABBBBBBB is “2 times [5 alternations and 5 repetitions]”. The code is available online at https://github.com/sromano/language-of-geometry.

Given this language of thought, for each sequence, one can find the simplest expression that describes it, and its associated complexity level (analogous to KC). Complexity is calculated by as a weighted sum of the fixed cost attached to each primitive instruction (+0, and b). As in our previous work [58], the additional cost for repeating an instruction *n* times is assumed to correspond to log_10_(*n*) (rounded up), i.e. the number of digits needed to encode the number in decimal notation. The relative value of those two costs is such that even a single repetition compresses an expression: +0^2 is assumed to be more compressed than the mere concatenation of +0+0 (see supporting information in [58] for details). As a result, the language favors an abstract description of sequences based on the maximum amount of nested repetitions, thus sharply dissociating sequence length and complexity. Among the multiple expressions that can describe the same sequence, the expression (or in some cases, the multiple expressions) with the lowest complexity is thought to correspond to the human mental representation of the sequence. In a nutshell, the assumption is that, in order to minimize memory load, participants mentally compress the sequence structure using the proposed formal language. The use of a minimal number of unitary operations, as well as the selection of the shortest representation, is in accordance with a simplicity principle, proposed as an essential component of learning, which states that the simplest hypothesis should be favored [32,55].The low impact of length on LoT complexity makes it markedly different from other metrics such as algorithmic complexity [43,44,46], for which longer sequences are systematically considered more complex since they are far less probable (even if longer by only one item). Although other complexity measures, such as change complexity, are correlated with ours, as further described below, they may also differ substantially for long sequences that can be hierarchically represented (e.g., AAABBB has the same LoT complexity as AAABBBAAABBB, since both are captured by a formula with two instructions and two digits, while change complexity is three times greater for the latter than for the former). Thus, the existing theories make distinct predictions, and it should be possible to empirically decide which one provides the best fit to human sequence memory abilities.

### Probing memory for sequences: The sequence violation paradigm

What is the best way to estimate such abilities? Previous research on sequence complexity has largely relied on either subjective judgments or explicit sequence reproduction in human adults [61]. Here, however, we required a more basic measure of sequence memory that did not require any language skills, explicit production of responses, and would therefore be generally applicable to human adults as well as, in the future, to infants and to non-human animals. Our approach consisted in assessing the capacity to detect rare violations in an otherwise regular sequential input. At the most elementary level, in the oddball paradigm, the simple repetition of an auditory or visual stimulus with a regular timing suffices for the brain to generate expectations, such that the unexpected violation of this regularity (e.g. AAAAB) gives rise to an automatic surprise or novelty response. Such a surprise effect can be detected behaviorally, e.g. using an explicit detection, a pupillary response, or electrophysiological signatures including the mismatch negativity [61–63], and it has been successfully used in non-human primates as a language-independent test of sequence learning [5,64,65].

A more complex brain response to novelty arises in the local-global paradigm [66,67], which contrasts two levels of violation: a local one, when a B stimulus follows a series of As (as in AAAAB); and a global one where, at a higher hierarchical level, the habitual sequence (e.g. AAAAB repeated multiple times) is replaced by a difference sequence (e.g. AAAAA). The use of this paradigm with neuroimaging made it for instance possible to show that macaques tend to spontaneously encode simple sequential patterns, using a cerebral network similar to the one in humans [13,65,68], or that such ability is already present in human infants [69]. It was also successfully used to show, with asleep participants or unconscious patients, that the processing of auditory sequential inputs at the global level (i.e. the level of patterns) is mainly restricted to conscious processing [66,70,71]. Behavioral and hemodynamic novelty responses to violations were also used by Huettel et al. [19] to show that human adults spontaneously encoded simple repeating and alternating patterns: categorisation response times and fMRI frontal activity patterns varied when such local patterns were violated (e.g. AAAAB or ABABB). Interestingly, the strength of the novelty response observed when the pattern was violated scale with the length of the preceding pattern (e.g. AAAAAB > AAAB), suggesting that the novelty response may perhaps track sequence complexity.

Here, we test the hypothesis that the violation detection task can be used to probe the encoding of sequences of higher level of complexity, thus revealing their degree of psychological regularity and providing insights into the internal language of thought used to encode them. By asking participants to detect when the presented sequence differed from the standard one (as presented multiple times during a habituation phase and throughout the experimental block), our experiments targeted a short-term memory process that, we argued, involves an internal compression as postulated in our LoT. We furthermore chose this paradigm with the aim of paving the way to future studies using non-verbal subjects or relying on brain measures of implicit violation detection.

### Statistical learning in sequence processing

A language of thought is by no means the only way to encode binary sequences. At a lower level of abstraction, the detection of sequential structures in the environment involves the identification of statistical regularities in the frequencies of items or the transitions between them [4,20]. Even in the language domain, transition probabilities are known to play an important role: eight-month-old infants have for instance been shown to rely on transition probabilities between syllables in order to segment a continuous stream of syllables into distinct words [72,73]. Transition probability learning, revealed by the observation of a novelty response to an improbable transition, was also reported in the visual modality [74,75], as well as in non-human primates [76,77]. This process appears to be automatic and continues to operate under non-conscious conditions [66,70,71]. When using novelty responses as an indicator of sequence complexity, it is therefore essential to separate the respective contributions of statistical learning and of a putative language of thought.

Computational models relying on probabilistic inference have been proposed for statistical learning. Mars et al. [78] for instance showed that the trial-by-trial modulation of the amplitude of a late novelty response, the P300, could be explained by a model tracking the frequency of individual items (among 4) in a temporal sequence. Similarly, our team proposed a Bayesian model for the acquisition of transition probabilities (not simply item frequency), and showed that it could explain a great variety of different behavioral and brain observations in binary sequence processing experiments [20,21]. The degree of confidence in a prediction can also be predicted using such a computational approach [79,80]. In these models, Shannon surprise, a mathematical measure of the improbability of a given item given the previous history of items [81–83], is a good predictor of behavioral and neural responses.

Thus, prior research indicates that, at a minimum, two distinct systems may underlie sequence learning in the human brain: statistical versus rule-based learning [4,66,84]. What is unknown is whether they operate independently and whether one is privileged at the expense of the other depending on the nature of the information to be encoded. We argue that any attempt to uncover the specific cognitive mechanisms behind rule learning in humans, especially in comparison with other species, must take into account the contribution of the less abstract yet powerful prediction system based on the statistical properties of events.

### The current study

In summary, our hypothesis was that, when confronted with a sequence, individuals spontaneously recode it in an abstract form, using an internal “language of thought” composed of a limited set of simple rules that can be hierarchically embedded. To test this hypothesis, we conducted a series of behavioral experiments in which participants were asked to listen to short auditory binary sequences (alternations of a sound “A” and a sound “B”), whose statistical properties and predicted complexity varied. We probed the participants’ ability to detect rare violations of the learned sequence (i.e. when one tone was replaced by another). Our hypothesis was that, for equal sequence length, error rate and response time in violation detection would increase with sequence complexity. In some experiments, in addition to those measures, we also asked participants to report subjective ratings of complexity. Finally, in one experiment, we compared auditory and visual sequences to assess whether our findings would extend to other sensory modalities.

For analysis, we examined the correlation between behavioral data and the shortest description length in the proposed language of thought (hereafter called LoT complexity to distinguish it from other complexity measures). To distinguish between rule-based and statistical learning mechanisms, we compared LoT complexity and Shannon surprise as predictors of performance. We started with long sequences of 16 items (experiment 1), and then probed the adequacy of the proposed language to shorter sequences (experiments 2–5). A simple prediction is that shorter sequences are more likely to be stored in a verbatim representation in working memory, without any internal compression. Thus, we predicted that the effect of LoT complexity in the proposed language of thought would increase as the sequence gets longer. On the other hand, given the automaticity of statistical learning, we did not expect any difference in its contribution to long versus short sequences. After examining the adequacy of the language for predicting task performance for each of the different experiments (with different lengths), analyses combining the data from multiple experiments were finally conducted, first to better assess the influence of complexity, length and transition probabilities in sequence processing, and second to compare the proposed LoT complexity to other computational approaches to sequence complexity proposed in the literature.

## Results and discussion

### Experiment 1: Auditory sequences with 16 items

**Fig 2:**
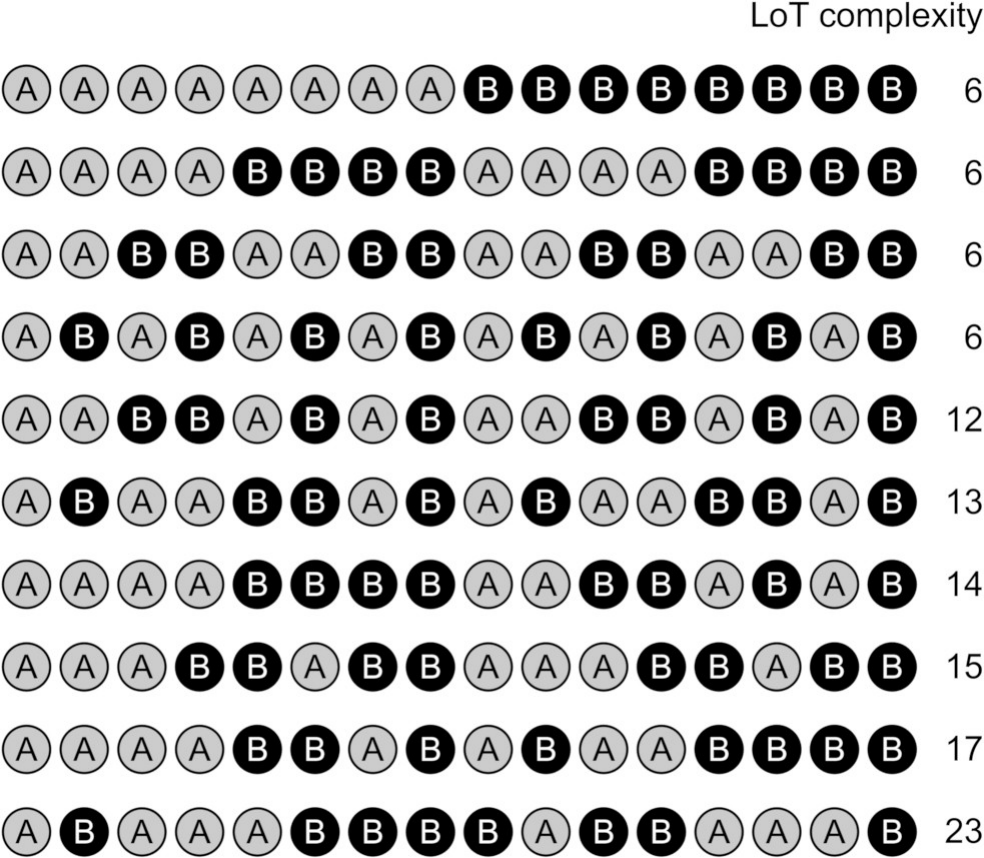
Ten 16-items long sequential patterns used in experiment 1, with their corresponding LoT complexity value.

In experiment 1, we selected 10 auditory sequences of 16 items, a number that vastly exceeds working memory capacity, which typically evolves between 4 to 9 items [25,29,85]. All sequences had equal numbers of sounds A and B (to reduce confounds related to the relative probability of As and Bs, thus controlling for stimulus-specific habituation effects), yet they varied widely in LoT complexity (see Fig 2). We obtained from subjects both subjective ratings of complexity and response times in response to deviants using a sequence violation paradigm (see Materials and Methods). Two types of violations were introduced: sequence deviants in which an A was replaced by a B or vice-versa; and “super-deviants”, in which an A or B was replaced by a rare novel tone C (see Fig 1B). We predicted that (i) the detection of sequence deviants would be affected by sequence complexity, because the detection of a deviant requires the encoding of the true sequence, and (ii) that the detection of deviants would be more difficult for more complex sequences. By contrast, super-deviants were not expected to yield a complexity effect, however, since they deviated from other stimuli at the most basic stimulus-frequency level. Super-deviant stimuli were introduced in an effort to ensure an invariant task which would equalize level of attention in all blocks, regardless of sequence complexity.

#### Complexity rating task

**Fig 3:**
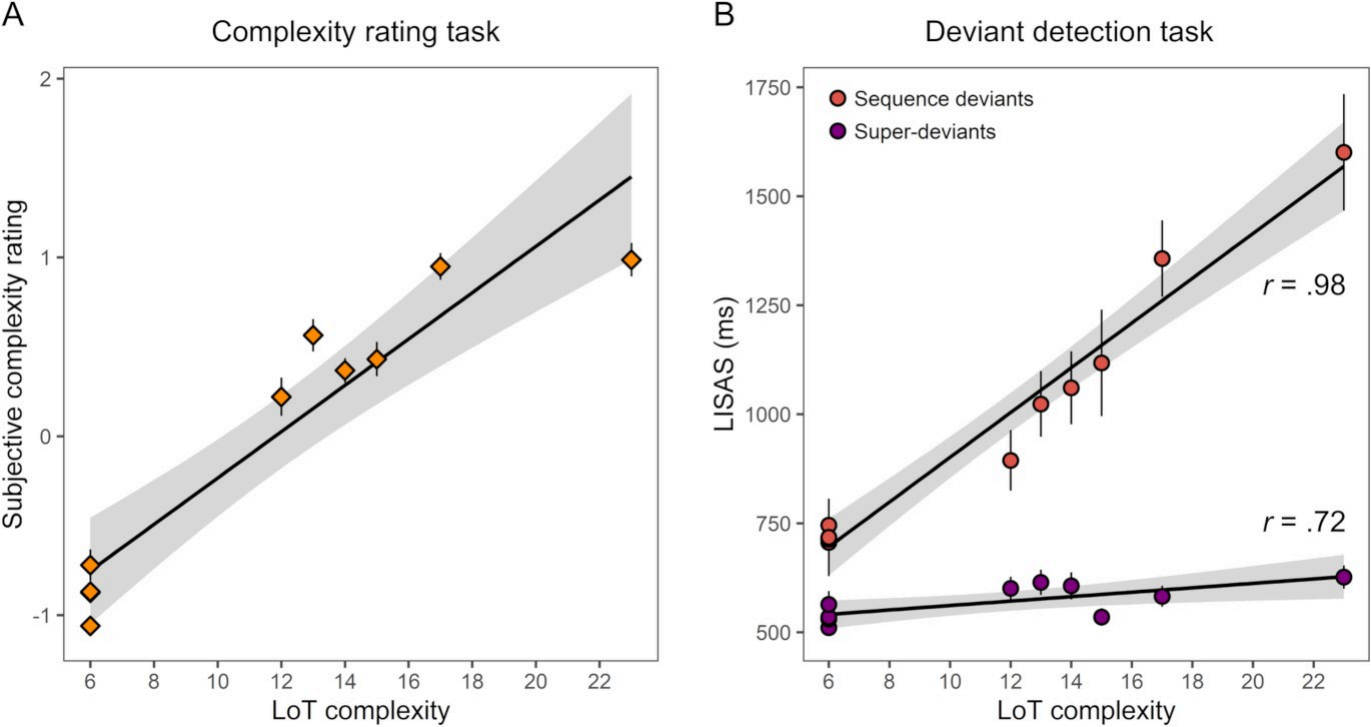
Linear relationship between LoT complexity and subjective and objective measures obtained in experiment 1 with ten 16-items long auditory sequences (with 95% confidence intervals bands in gray). The Pearson correlation coefficient (*r*) is indicated. Each marker represents the group-average for a given sequence. Error bars represent SEM across participants. (A) LoT complexity vs. subjective complexity ratings. (B) LoT complexity vs. performance in the violation detection task (Linear Integrated Speed-Accuracy Score), for sequence deviants and super-deviants.

We observed a strong positive linear relationship between average subjective complexity ratings and LoT complexity (entered as a fixed factor in the linear mixed model including participants as the random factor: *t*(278) = 24.6, p < .0001; Pearson correlation coefficient on the average ratings for each sequence: *r* = .94) (see Fig 3A). These results indicate that participants were readily able to judge whether a pattern is “more complex” than another, and that the formal language we used to compute sequence complexity is close to how individuals form such complexity judgements.

#### Deviant type and complexity effects in the violation detection task

We observed a linear relationship of LoT complexity and performance in the violation detection task (using the *Linear Integrated Speed-Accuracy Score*, LISAS, an integrated measure of response times and error rates, see [86,87]). We observed main effects of LoT complexity (*t*(415.0) = 18.1, p < .0001), deviant type (994 ms for sequence deviants vs. 570 ms for super-deviants; *t*(414.4) = 18.9, p < .0001) and their interaction (*t*(414.5) = 11.7, p < .0001). Indeed, the slope of the complexity effect was significantly stronger, by an order of magnitude, for sequence deviants as opposed to super-deviants (respectively +51 ms vs. +5 ms in simple regression, *t*(16) = 11.7, p < .0001; see Fig 3B and S1 Fig for the corresponding results using response times or miss rate instead of LISAS). Nevertheless, separate analyses revealed that LoT complexity was a strong predictor of performance for sequence deviants (*t*(193.0) = 15.5, p < .0001; *r* = .98) and also, surprisingly, for super-deviants (*t*(198.5) = 4.08, p < .0001; *r* = .72) (Fig 3B). The latter effect on LISAS was however mainly driven by response times, since the average hit-rate for super-deviants was high (96%) and weakly modulated by LoT complexity (*t*(200.7) = 2.32, p = .022).

The number of false alarms per sequence (which was 1.99 on average) also increased with sequence LoT complexity (*t*(214.4) = 4.20, p < .0001; *r* = .74), suggesting here again that the LoT complexity was a good predictor of the quality of sequence encoding.

The results of this first experiment with long binary auditory sequences (16 items) thus indicate that the formal language used to describe sequences in a compressed form, based on simple (possibly embedded) rules, is highly relevant to predict (i) how “complex” an auditory sequence is judged by adult participants after having listened to it once and (ii) how difficult it was to learn these sequences in order to detect alterations.

Sequence complexity was expected to have little or no impact on the detection of super-deviants, i.e. high or low pitch tones different from the two tones composing the binary auditory sequence. Our rationale was that such “C” tones were detectable even without any prior knowledge of sequence structure. While performance in detecting super-deviants was much better than for sequence deviants, even for the simplest sequences, a clear relationship between LoT complexity and performance continued to be observed. We see at least two interpretations of this finding. First, there could be an increased attentional cost of having to detect violations in more complex sequences, thus placing subjects in a dual-task setting of having to simultaneously maintain a complex representation in memory and to respond to deviants. Alternatively, the effect could reflect the influence of a top-down prediction system which would use sequence structure to generate predictions of the incoming stimuli. Complex sequences would be less well predicted, and this would in turn affect the speed with which any deviant is detected. We return to this question in the *General Discussion*.

#### Surprise effects

Many prior experiments, using either or both behavior and brain-imaging measures, have shown that individuals constantly entertain predictions about future observations using probabilistic knowledge based on past observations [e.g. 20,21]. In order to test whether task performance could be explained by a learning transition probabilities (surprise) only, or also truly implied an encoding of sequence structure, we compared a mixed model (with participants as a random effect) including fixed effects of both LoT complexity and surprise (averaged across the 4 possible positions of deviants in a given sequence) with a null model including only surprise. The effect of surprise in the null model with surprise alone) was significant (*t*(193.0) = 5.31, p < .0001). However, a likelihood ratio test showed that adding LoT complexity significantly improved the goodness of fit: χ^2^(1) = 130.9, p < .0001. Adding a “period” factor (i.e. period values were 2, 4, 8 or 16) as a third fixed effect did not improve the model fit (χ^2^(1) = 1.23, p = .267), confirming the prediction that the four included A^*n*^B^*n*^ patterns have the same psychological complexity, and suggesting that this information is already captured by LoT complexity. Adding the interaction between surprise and LoT complexity did not improve goodness of fit either (χ^2^(1) = 2.50, p = .114). As reported in Table 1, the LoT complexity fixed effect was significant in the final full model (*t*(192.4) = 13.6, p < .0001), but not the surprise fixed effect (*t*(191.8) = 0.60, p = .55). The absence of a significant effect of surprise once sequence complexity is taken into account reflects the existence of a correlation between the two measures (*r* = –.54): biased transition probabilities in less complex sequences tending to make deviants more easily surprising. It also shows that when these two slightly colinear factors are included, LoT is more effective than surprise at describing the variance of the data.

As our choice of attributing an arbitrary padding value (0.01) to deviant transitions events with zero probability when computing surprise may have biased the results, we recomputed the LISAS and average surprise while excluding all such trials (i.e. all deviant positions in the (AB)^8^ pattern, 3 out of 4 deviant positions in the A^8^B^8^ pattern). Here again, a likelihood ratio test showed that the goodness of fit increased significantly when adding LoT complexity to a null model containing only surprise (χ^2^(1) = 116.3, p < .0001). However, both complexity (*t*(165.5) = 12.9, p < .0001) and surprise (*t*(165.8) = 3.82, p < .0001) were significant with this subset of the data.

In conclusion, the strong complexity effects observed here indicated that participants used some form of compression of information to encode the sequence and perform the task over and above simply learning statistical trends. Although no instruction was given in that sense, this strategy may be needed in order to deal with a difficult, memory-demanding task. Indeed, at the maximum level of complexity used, performance in violation detection was very low (the violation detection rate dropped to 41% for sequence deviants).

In the subsequent experiments, we asked whether similar complexity effects emerged in the same paradigm but with shorter sequences. That is, when the sequence can be more easily encoded and stored “as a whole”, without necessarily requiring a re-encoding in a more abstract, compressed form. In these less demanding conditions, it can be expected that the spontaneous encoding of transitions probabilities between items will play a more important role in the detection of violations.

### Experiment 2: Auditory sequences with 12 items

**Fig 4:**
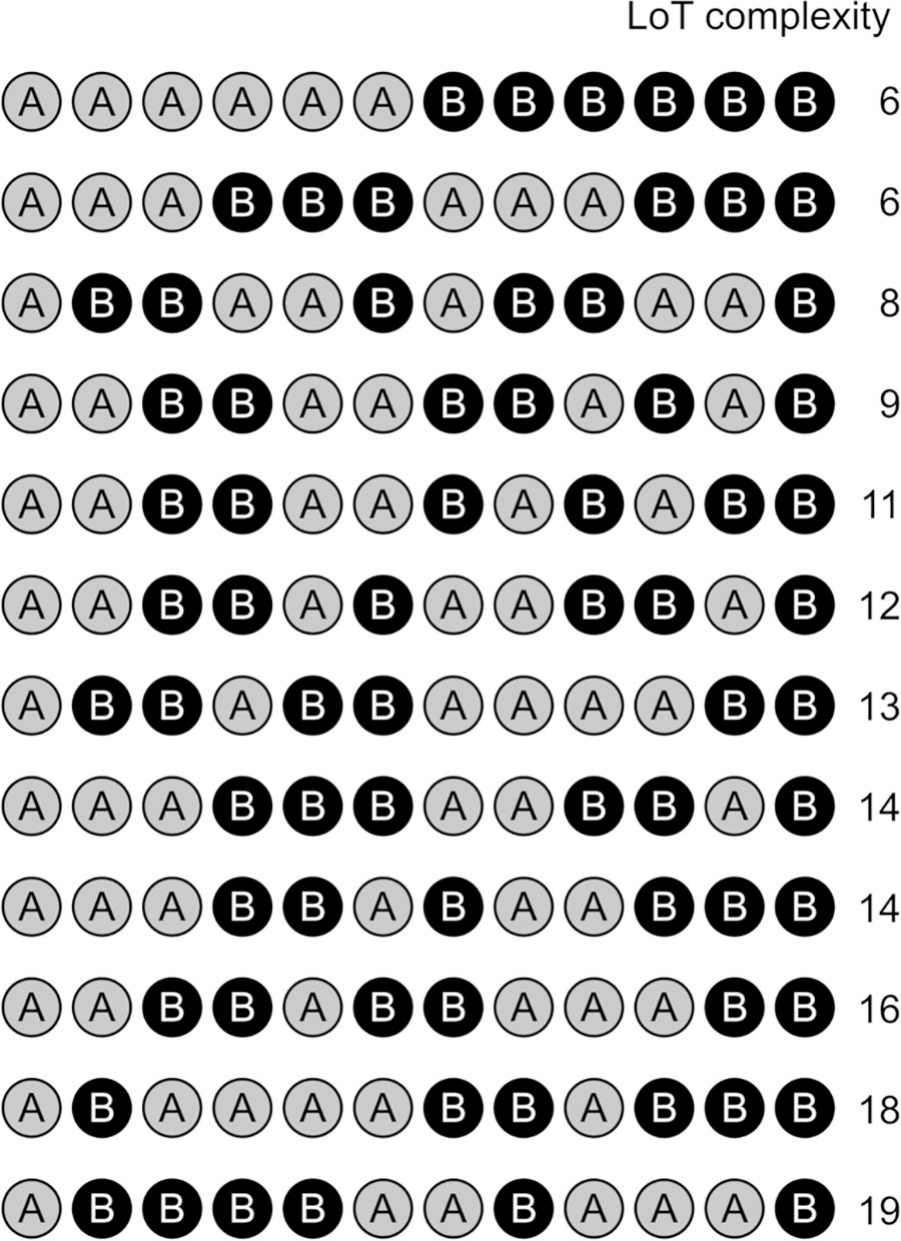
Twelve 12-item sequences used in experiment 2, with their corresponding LoT complexity value (in bits).

In order to test whether the previous results could be replicated with shorter sequences, in experiment 2, the same tasks and procedure were used (with a different group of participants), this time using twelve sequences of twelve items (spanning a large range of complexities, see Fig 4).

#### Complexity rating task

**Fig 5:**
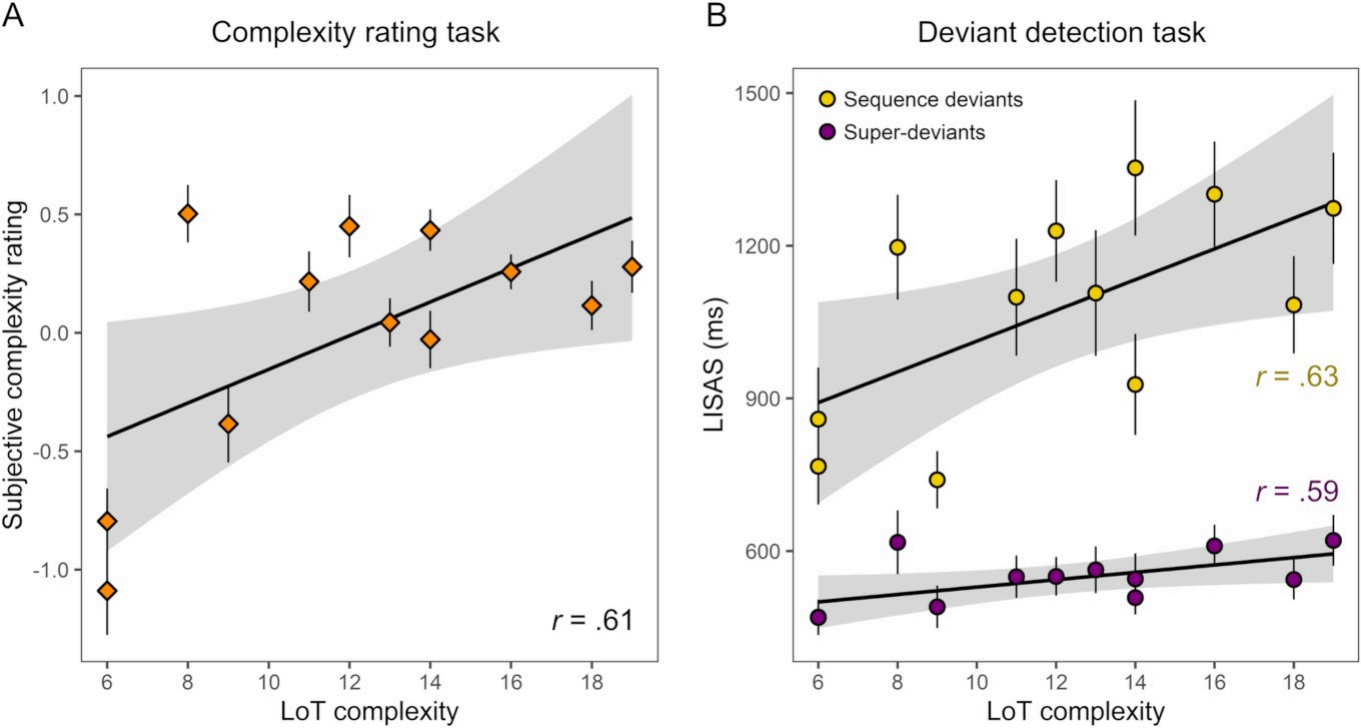
Linear relationship between LoT complexity and scores obtained in the two tasks of experiment 2 with 12-item auditory sequences (with 95% confidence intervals bands in gray). Same format as Fig 3.

A positive linear relationship was found between subjective complexity ratings and LoT complexity (*t*(238) = 6.81 p < .0001, *r* = .61). The correlation of the average score per sequence with LoT complexity was however less strong than what was observed in the previous experiment with 16-items long sequences (*r* = .61, see Fig 5A). Subjective complexity was clearly underestimated for one specific sequence (ABBAABABBAAB, predicted complexity of 8), which is confirmed by an inspection of the residuals of the regression (residual 1.99 *SD* above average for this sequence).

### Deviant type and complexity effects in the violation detection task

Regarding the violation detection task, main effects of LoT complexity (*t*(431.1) = 6.43, p < .0001) and deviant type (1078 ms for sequence deviants vs. 545 ms for super-deviants; *t*(431.0) = 19.3, p < .0001) were observed, as well as their interaction (*t*(431.1) = 3.48, p < .001). The slope of the complexity effect appeared indeed slightly stronger for sequence deviants as opposed to super-deviants, although the comparison did not reach significance when using simple linear regressions with averaged LISAS per sequence (slopes of respectively +30 ms vs. +7 ms, *t*(20) = 1.87, p = .077; see Fig 5B and S2 Fig for the corresponding results with RTs and miss rates instead of LISAS). Separated analyses revealed that the effect of LoT complexity was significant in analyses restricted to either sequence deviants (*t*(205.1) = 5.78, p < .0001; *r* = .63), or super-deviants (*t*(208.0) = 2.88, p = .005; *r* = .59) only. The number of false alarms per sequence (3.88 on average) was also significantly predicted by the LoT complexity of the sequence (*t*(208.0) = 3.50, p < .001; *r* = .56).

As in the complexity rating task, although the overall correlation was high, a noticeable deviation between predicted complexity and observed performance was present for some of the sequences. In fact, the correlation profiles observed in the Fig 5A and 5B suggest that the psychological complexity of the pattern, as indexed by subjective rating or violation detection task performance, might have been, for some sequences, consistently overestimated or underestimated by the LoT across both tasks (the largest residual in the regression with the sequence deviants, 1.50 *SD* above average, corresponded to the same sequence identified by complexity ratings: ABBAABABBAAB). To further test this idea, we computed the correlation between the residuals of both linear regressions. The correlation was significant (*t*(10) = 4.02; p = .003), indicating that even after regressing out the effect of LoT complexity, the data from both experiments remained correlated with each other, and thus that, although the proposed LoT is a good predictor, it does not fully account for all details of the psychological complexity of patterns. One attempt to address the limitations of the language, by proposing a modification of it, is reported in the *Further analysis* section.

#### Surprise effects

A comparison of mixed models (with participants as a random effect) showed that, compared to a null model including surprise as the sole predictor (null model; in which the main predictor was significant: *t*(205.0) = 4.67, p < .0001), a model additionally including LoT complexity (full model) fitted the data better (likelihood ratio test: χ^2^(1) = 14.4, p < .001). Both fixed effects were significant in the full model: LoT complexity (*t*(204.1) = 3.85, p < .0001), as well as surprise (*t*(204.0) = 2.05, p = .042) (see Table 1). Although we observed, contrary in the previous experiment, an effect of statistical learning (indexed by the level of surprise of deviant items), it was only barely statistically significant.

**Table 1:** *Fixed effects in the linear mixed models separately for each experiment*.

**Experiment 1** (16-items sequences, excluding super-deviants)					
*Predictors*	*Estimates*	*Std*. *Error*	*T-value*	*95% CI*	*p*
(Intercept)	356.90	80.51	4.43	199.5–514.3	**< .0001**
Complexity	52.15	3.84	13.60	44.6–59.7	**< .0001**
Surprise	6.77	11.31	0.60	-15.4–28.9	.55
**Experiment 2** (12-items sequences, excluding super-deviants)					
*Predictors*	*Estimates*	*Std*. *Error*	*T-value*	*95% CI*	*p*
(Intercept)	852.38	124.91	6.82	608.5–1096.2	**< .0001**
Complexity	24.21	6.29	3.85	11.9–36.5	**< .0002**
Surprise	-43.13	21.06	-2.05	-84.4 –-1.9	**< .05**
**Experiment 3** (8-items sequences)					
*Predictors*	*Estimates*	*Std*. *Error*	*T-value*	*95% CI*	*p*
(Intercept)	852.40	73.39	11.62	707.8–997	**< .0001**
Complexity	10.75	3.49	3.08	3.9–17.6	**< .003**
Surprise	-32.37	5.60	-5.78	-43.3 –-21.4	**< .0001**
**Experiment 4** (6-items sequences, sequence 'AAAAAA' excluded)					
*Predictors*	*Estimates*	*Std*. *Error*	*T-value*	*95% CI*	*p*
(Intercept)	751.6	47.5	15.8	658.8–844.5	**< .0001**
Complexity	1.4	4.4	0.3	-7.2–9.9	.75
Surprise	-15.3	3.8	-4.1	-22.7 –-7.9	**< .0001**
**Experiment 5** (8-items sequences, auditory and visual)					
*Predictors*	*Estimates*	*Std*. *Error*	*T-value*	*95% CI*	*p*
(Intercept)	645.1	92.2	7.0	464.4–825.9	**< .0001**
Complexity	25.2	25.2	4.4	14–36.4	**< .0001**
Surprise	-36.7	8.1	-4.5	-52.5 –-20.8	**< .0001**
Modality (Visual)	337.0	337.0	14.2	290.7–383.3	**< .0001**

### Experiment 3 and 4: Auditory sequences with 6 or 8 items

Results of experiments 1 and 2 showed that our sequence complexity metric was well correlated with behavior, suggesting that our formal language provided a good approximation of the internal language of thought that humans use to encode a sequence in memory a compressed from. These results were however obtained with a restricted set of sequences, which were long enough to promote hierarchical representations based on the repetition and alternation operations, and to probe a large range of complexity values. The main objective of experiments 3 (with 35 8-items long sequences, see S3 Fig) and 4 (with 32 6-items long sequences, see S4 Fig) was to test whether the effect of complexity observed in the first two experiments could be generalized to a larger set of shorter sequences, where we could examine more gradual variations in complexity. Given that human working memory is thought to store and maintain 4 to 7 items without compression, or with a minimal chunking process [25,29], we expected the predictive power of our language to be reduced compared with previous experiments with longer sequences, while the effects of transition probabilities would increase. The same violation detection paradigm was used. No subjective complexity ratings were collected (given the larger number of individual sequences compared to the previous experiments).

**Fig 6:**
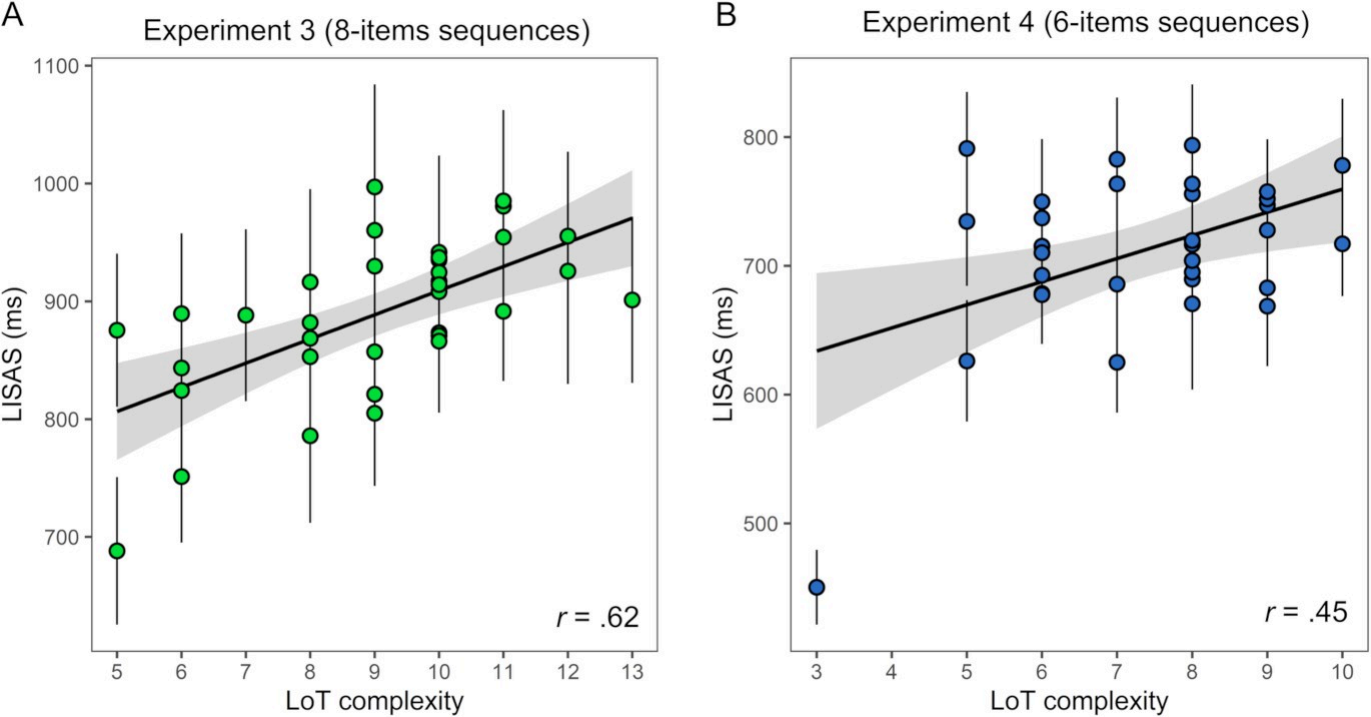
Linear relationship between LoT complexity and violation detection task performance (LISAS) in: (A) experiment 3 (8-items sequences) and (B) experiment 4 (6-items sequences).

Here again, we tested (using mixed models) whether surprise suffices to explain the variance in performance or if a significant proportion of variance remained yet to be explained by sequence complexity (all models included participants as a random effect). In experiment 3 (8-items sequences, *N* = 35), goodness of fit improved when LoT complexity was included in the model (χ^2^(1) = 9.47, p = .002). Both fixed effects were significant in the full model: LoT complexity (*t*(1042.0) = 3.08, p = .002; see Fig 6A and S5 Fig), as well as surprise (*t*(1042.0) = 5.78, p < .0001) (see Table 1). Note that the surprise fixed effect was already highly significant in the null model (*t*(1043.0) = 8.72, p < .0001).

Similarly, in experiment 4 (6-items sequences, *N* = 32), goodness of fit improved when LoT complexity was included in the surprise-only null model (χ^2^(1) = 6.20, p = .013) with both fixed effects significant in the full model (LoT complexity: *t*(649.00) = 2.49, p = .013; see Fig 6B and S6 Fig), and surprise (*t*(649.0) = 5.48, p < .0001). The surprise fixed effect was here again already highly significant in the null model (*t*(650.0) = 6.78, p < .0001). However, one sequence appeared as an outlier in this experiment, with an average LISAS 3.9 *SD* below the average of all sequences (i.e. indicating a much better performance): the AAAAAA sequence. In this case, performing the task requires no sequence learning, but merely remembering the identity of the A sound, and violation detection is therefore similar to a classic oddball paradigm. When this sequence was removed from the dataset (it was also excluded from further analyses), the inclusion of the complexity fixed factor did no longer improved model goodness of fit (χ^2^(1) = 0.10, p = .752). Indeed, the LoT complexity fixed effect was not significant in the full model (*t*(628.0) = 0.32, p = .752), as opposed to the surprise fixed effect (*t*(628.0) = 4.07, p < .0001) (see Table 1). No improvement in model fit was found when including the interaction between complexity and surprise (χ^2^(1) = 0.08 in experiment 3, χ^2^(1) = 0.34 in experiment 4).

Beside the effect of complexity, the strong effect of surprise in both experiments indicates that participants were quicker and more likely to detect a deviant when it violated statistical regularities characterizing the auditory sequence being repeatedly played. This is consistent with the idea that humans spontaneously encode the probabilities associated with events and react to surprising events depending on their level of predictability [19,21].

The number of false alarms was low in the present experiments (0.91 per sequence on average in experiment 3, 0.60 in experiment 4). It was slightly related to sequence complexity in experiment 3 *t*(1048) = 2.19, p = .029) but not in experiment 4 *t*(650.0) = 0.29, p = .77).

Compared to the previous experiment with lengths 12 and 16, it was expected here, with sequences of 8 or 6 items, that the effect of LoT complexity would be mitigated, since those auditory sequences may become short enough to be stored in working memory as a simple chain (note that the range of LoT complexity values was also smaller). The correlation of performance with LoT complexity was in fact still present with 8-items sequences (at a similar level as in experiment 2) but disappeared with 6-items sequences. This is in line with the assumption that complexity is tightly linked with the idea of compressibility in memory, and suggests that such a compression strategy, whether it is simple chunking or involves a hierarchical representation, is more likely to be involved when the number of items to store in working memory exceeds the typical working memory span [16,88]. However, rather than a clear threshold above which complexity would become predictive of performance, the estimates of the LoT complexity effect across the four experiments (in the mixed models taking into account surprise) reveal a gradient: with stronger effects of complexity for longer sequences (respectively +1.4 ms, +10.8 ms, +24.2 ms, and +52.2 ms, for the experiments with length 6, 8, 12 and 16 respectively; see Table 1). The effect of surprise seemed to follow an inverse trend, with insignificant or marginal effects in long sequences (experiments 1 and 2) and highly significant effects in short sequences (experiments 3 and 4). To test this idea, the data from experiments 1–4 (excluding super-deviants) were combined in a single mixed model including the three fixed factors of LoT complexity, surprise and length (as a continuous predictor), as well as the three two-way interactions (with participants as the random factor). An ANOVA on the mixed model revealed main effects of LoT complexity (*F*(1, 2336.4) = 48.0, p < .0001) and surprise (*F*(1, 2334.1) = 4.91, p = .027). The main effect of sequence length was marginally significant (*F*(1, 96.6) = 3.08, p = .082). As expected, a strong interaction between LoT complexity and length was present (*F*(1, 2347.5) = 63.3, p < .0001), indicating a stronger effect of complexity when sequence length increased. The estimated slopes for the LoT complexity effect indeed increased with each sequence length (+15.5 ms, +46.0 ms, +107.1 ms, and +168.1 ms, for length 6, 8, 12 and 16, respectively). The interaction between length and surprise was not significant (*F*(1, 2330.0) = 1.19, p = .276). However, the estimated slopes for the surprise effect followed our initial observation: they decreased with each sequence length (-15.6 ms, -12.0 ms, -4.9 ms, and +2.2 ms).

### Experiment 5: Auditory and visual sequences

The observation of a LoT complexity effect on sequences of length 8 and higher is consistent with our initial claim that individuals spontaneously apply simple rules (mainly based on nested repetitions) in order to recode auditory sequences in a compressed abstract form in memory. It may be argued, however, that rather than being abstract and universal, some of these effects may reflect the great ability of our auditory system to manipulate and find regularities in acoustic stimuli [89]; whether it is in spoken language or in music listening. In experiment 5, we wished to replicate the findings of previous experiment and extend them to the visual modality. Although we expected a reduced performance, given that audition is generally superior to vision in the processing of temporal information [90], we still predicted a correlation of performance with our complexity metric, since our language was originally designed for a visual paradigm [58] and relies on abstract mental operations rather than on specific acoustic coding mechanisms. Twelve sequences of 8 items (see S7 Fig), allowing to use a sufficient number of trials while still expecting clear complexity effects, were presented to a group of participants in both a visual and in an auditory form (in different experimental blocks), using the same violation detection paradigm. Due to constraints in the perception of repeated visual stimuli, stimulus onset asynchrony was lengthened to 400 ms in both auditory and visual sessions, resulting in a sequence duration of 3000 ms (compared to 1800 ms in experiment 2).

#### Complexity and modality effects

**Fig 7:**
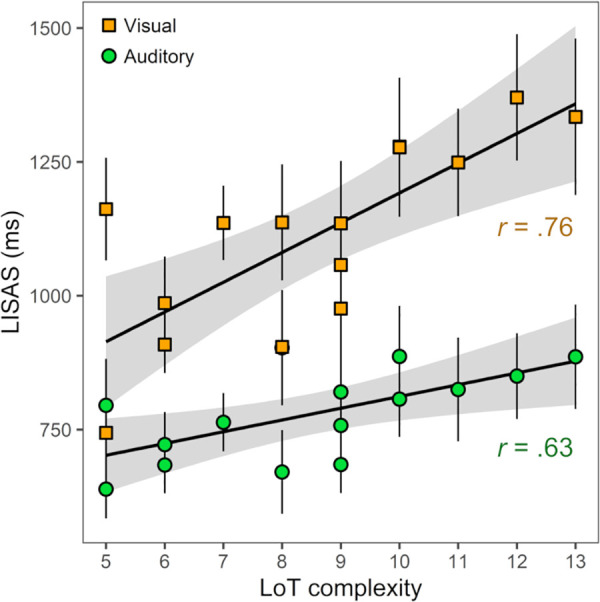
Linear relationship between LoT complexity and violation detection task performance (LISAS) for each modality in experiment 5 (8-items auditory and visual sequences).

To assess the impact of LoT complexity and modality on performance, we first estimated a mixed model including complexity and modality as fixed factors and participants as a random factor. Effects of LoT complexity (*t*(486.0) = 3.08, p = .003), modality (average LISAS of 1110 ms in visual blocks vs. 780 ms in auditory blocks; *t*(486.0) = 14.1, p < .0001) and their interaction (*t*(486.0) = 3.19, p = .002) were significant. The slope of the complexity effect was steeper in the visual than in the auditory modality (+54 ms vs. +22 ms, *t*(486) = 3.19; see Fig 7 and S8 Fig). Separate analyses indicated that LoT complexity was a strong predictor of performance for visual sequences (*t*(233.0) = 6.82, p < .0001; *r* = .76), and also for auditory sequences (*t*(237.0) = 3.76, p < .001; *r* = .63).

Note that, although the effects appeared stronger in the visual modality, the average performance in the visual and the auditory modality were highly correlated (*r* = .85, p < .0001). This suggests a common, cross-modal mechanism underlying the observed differences in performance between sequences. It can however be acknowledged, here again, that differences in performance across sequences are not entirely explained by complexity: residuals of linear regressions with LoT complexity in the visual and in the auditory modality (using average LISAS per sequence) were correlated (*r* = .73, *t*(13) = 3.92; p = .002).

The number of false alarms per sequence was related to the task modality (mean number of FA: 0.58 in auditory blocks; 1.16 in visual blocks; difference between modalities: *t*(487.0) = 5.73, p < .0001) but not to sequence LoT complexity (*t*(487.0) = 0.08, p = .935).

#### Surprise effects

As in previous experiments, a surprise effect was also observed in both modalities when considered independently: deviants inducing rare transitions were more easily and quickly detected than frequent ones (effect of surprise in a mixed model with auditory trials only: *t*(237.0) = 3.87, p < .001; *r* = –.65; with visual trials only: *t*(233.0) = 6.79, p < .0001; *r* = –.78). This effect suggests that a common, or at least similar, mechanism is at play in the encoding of statistical regularities characterizing the sequences in both the visual and the auditory modality.

In order to test whether evidence for sequence compression could still be observed after the surprise effect was taken into account, we performed a comparison of mixed effects models. The null model included the surprise predictor, the modality as a categorical predictor and subject identity as random factor. It was compared against a full model including the same predictors, with addition of the LoT complexity. This comparison was highly significant (χ^2^(1) = 19.0, p < .0001), indicating that goodness of fit improved when LoT complexity was added to the model. All three fixed effects were significant in the full model (LoT complexity: *t*(486.0) = 4.39, p < .0001; surprise: *t*(486.0) = 4.54, p < .0001; modality: *t*(486.0) = 14.2, p < .0001, see Table 1).

Overall, the results obtained in the visual modality are very similar to those obtained in the auditory modality in the same and in previous experiments. We however observed here stronger effects of both LoT complexity and surprise. It should be noted that the overall difficulty of the task increased in the visual modality (as indicated by higher average miss rates per sequence; 22% vs. 11%, *t*(14) = 7.49, p < .0001; and longer average response times per sequence; 831 ms vs. 645 ms, *t*(14) = 10.5, p < .0001). 8-items visual sequences may have been more difficult to encode than 8-items auditory sequences, due to the known superiority of the auditory processing system in the processing of temporal sequences and rhythms [89,91]. This increased encoding difficulty in the visual domain may have in turn lead to an increased need for the “mental sequence compression” mechanism that our language of thought aims to describe.

The present experiment also extends the results of experiment 3 by using a slower presentation rate. Indeed, although the participants in experiment 5 appeared to respond faster (in the auditory blocks) than those from experiment 3, the same relationship with complexity was found (correlation of performance with LoT complexity of .62 and .63 respectively). It suggests that the effect of complexity is robust across sequence durations (as expected given than LoT complexity is based on abstract sequence patterns). More importantly, the fact that a similar complexity effect was observed irrespective of the modality is consistent with the idea of “language of thought” used to compress sequential information at an abstract, symbolic level. Such an assumption has already been supported by results from Yildirim and Jacobs [92], who showed cross-modal transfer of sequence knowledge: learning to categorize visual sequences facilitated the categorization of auditory sequences and vice versa. In fact, the language we used here was initially designed to represent visually presented, geometrical patterns [58]. The present results thus confirm that the present language of thought can account for sequence representations in various modalities, presentation contexts and sequence lengths.

### Further analysis: Comparison with other measures of sequence complexity

The complexity, or “compressibility”, of a sequence can be assessed in several ways, and various measures have been previously proposed in the psychological literature [e.g. 16,18,35,44,47,93–96]. In this last section, we examined how our LoT complexity value compares to six other measures, which we list below, in predicting task performance over different sequence lengths.

**Chunk complexity**: following the observation that the number of chunks (or runs) is correlated to performance in sequence encoding tasks [e.g. 35], we here define chunk complexity using the formula proposed by Mathy & Feldman [16], which they showed to correlate with performance in the encoding of series of digits: $Chunk\;complexity=\sum_{i=1}^{K}{log}_{2}(1+L_{i})$, where K is the number of chunks and L_i_ the length of the *i*-th run. Note that contrary to Mathy & Feldman [16], whose sequences where composed of digits and chunks defined based on constant (positive or negative) increments from one digit to the next (e.g. “1234”, “7531”), we here simply define chunks as consecutive repetitions of the same item, e.g. the sequence “AAABAA” has a 3 chunks, and a chunk complexity of *log*_*2*_*(4) + log*_*2*_*(2) + log*_*2*_*(3)*.

**Entropy** is a measure of information that quantifies the uncertainty of a distribution. Here, we compute the Shannon entropy of the probability of pairs of items, (AA, AB, BA, BB), in order to capture the effect of order-1 transition probabilities [84]. Given that the probability of a given pair is defined as *p*(*X,Y*) = *p*(*X*)∙*p*(*Y|X*), *H* is computed as follow:

$$H=-[p(A)∙p(A|A)∙({log}_{2}p(A)+{log}_{2}p(A|A))+p(A)∙p(B|A)∙({log}_{2}p(A)+{log}_{2}p(B|A))+p(B)∙p(A|B)∙({log}_{2}p(B)+{log}_{2}p(A|B))+p(B)∙p(B|B)∙({log}_{2}p(B)+{log}_{2}p(B|B))]$$

We used the convention that 0 × log_2_(0) = 0 when null probabilities occurred.

**Lempel-Ziv complexity** is derived from the popular lossless data compression algorithm, the Lempel-Ziv (LZ) algorithm [97]. Briefly, the LZ algorithm works by scanning the sequence from left to right and adding to a vocabulary each new substring it has never encountered before. LZ complexity is the number of substrings in this vocabulary once the scan is complete. Beyond the field of computer data compression, LZ complexity has been used in various domains, for instance, to measure the complexity of rhythmic patterns in music [98], to account for the complexity of human [99] and mouse behaviors [100], to explain the existence of universal properties within all natural languages [101], or to measure the complexity of input-output mappings found in various domains of science and engineering [102,103].

The number of **subsymmetries** is the number of symmetric sub-sequences of any length within a sequence. For instance, the sequence AABBAB has two symmetric sub-sequences of length 2 (AA and BB), one of length 3 (BAB), and one of length 4 (ABBA), for a total of four subsymmetries. This measure was proposed by Alexander and Carey [93] and shown to be negatively correlated to performance in perception and production tasks with visual and auditory patterns [93,104].

**Change complexity** is an measure proposed by Aksentijevic and Gibson [47], based on the notion of “change” (the inverse of invariance), computed across all sub-sequences contained in a sequence, and showing interesting properties such as a sensibility to periodicity and symmetries.

**Algorithmic complexity** was introduced by Gauvrit et al. [44,45] and Soler-Toscano et al. [46]. It is based on the mathematical definition of Kolmogorov-Chaitin complexity [39,40] and derived from the probability of obtaining a given pattern in the output of a randomly chosen universal Turing machine that halts.

**LoT chunk complexity.** Note that the alternative measures of complexity tested here, which provide a unique metric for each pattern, are conceptually quite different from the one we propose. LoT complexity is based on the proposal that humans possess a language of thought, composed of a small number of atomic rules which they use recursively to recode the abstract structure of the pattern in a compressed form. Such a recursive representation differs radically from, say, the mere counting of the number of chunks. However, it is possible to combine the two ideas. The formal language we proposed produces many legal expressions for each sequence (the number of possible expressions can reach several tens of thousands for a sequence of length 16), which correspond to distinct “parses” of the same sequence. We initially assumed that the shortest expression is always selected (with the limitation that two or more expressions can have the same “shortest” length for some sequences), and thus that LoT complexity is equal to the shortest possible description using this language. However, it is unclear whether humans could ever search such a vast space of possibilities. A more plausible hypothesis is that participants begin by chunking the sequence into groups of identical items, and only then compress it by detecting repetitions of those chunks [for a similar proposal, see 33,49]. According to this idea, the shortest sequence should only be accepted when its proposed parsing coincides with chunk boundaries. Consider the sequence ABBAAB, which consists of 4 chunks [A] [BB] [AA] [B]. According to our language, its optimal description is [AB] [BA] [AB] (i.e. 3 repetitions of the stay-change program; LoT complexity = 5), but that representation does not coincide with chunk boundaries. Interestingly, the data suggested that the shortest description may not be the best in similar cases (see *Experiment 2*, *Results and discussion*). To test this idea, we recomputed LoT values restricted to chunk-preserving expressions (i.e. excluding expressions producing “A][A” or “B][B”). We called this new LoT complexity the **LoT chunk complexity.** Its value was higher than the original one for 58% of sequences (and remained the same for the others). For instance, the sequence “ABBAAB” from the previous example, when described as four chunks [A] [BB] [AA] [B], has an LoT-chunk complexity = 9. We tested LoT chunk complexity as another potential predictor of behavioral performance.

#### Model comparison

With the aim of arbitrating between previous models, we pooled data from all previous experiment with auditory sequences (using LISAS to index task performance), excluding super-deviants. Unfortunately, due to the nature of algorithmic complexity (derived from the output frequency for a pattern using small Turing machines, which decreases rapidly with sequence length), no values were available for the ten length-16 patterns that we used in experiment 1, as well as for one length-12 pattern used in experiment 2. Those sequences were therefore excluded from some analyses. The sequence AAAAAA from experiment 4 was also excluded. Consequently, a first pooled dataset, for which all 8 different predictors could be compared, included performance with 77 different auditory sequences (and 88 different participants), of length 6 (*N* = 31 sequences), length 8 (*N* = 35) and length 12 (*N* = 11), while a second one, for which 7 different predictors were compared, also included sequences of length 16 (*N* = 88 sequences, 113 participants).

To assess whether one measure was a better predictor of task performance, we first computed different mixed models, which all included the predictor of interest as the only fixed effect and participants as a random effect (note that this is a way to control for the fact that different participants coming from different experiments, with different sets of stimuli, were pooled together). We then report the Akaike information criterion (AIC) as an indicator of goodness of fit which penalizes for model complexity (i.e. the number of predictors); the model with the lowest AIC value being considered the best (or with lowest Δ(AIC) value, i.e. the relative difference in AIC with the best model: for a model *i*, Δ(AIC)_i_ = AIC_i_—AIC_min_). Note that we also report the Bayesian information criterion (BIC) which, in addition, scales the strength of penalization by the (log) number of data points [105]. Second, since, as we reported earlier, surprise derived from the learning of transition probabilities may strongly affect the performance in such violation detection task, all these models were estimated again, this time including surprise as a fixed effect covariate.

#### Dataset with sequences of length 6, 8 and 12

**Fig 8:**
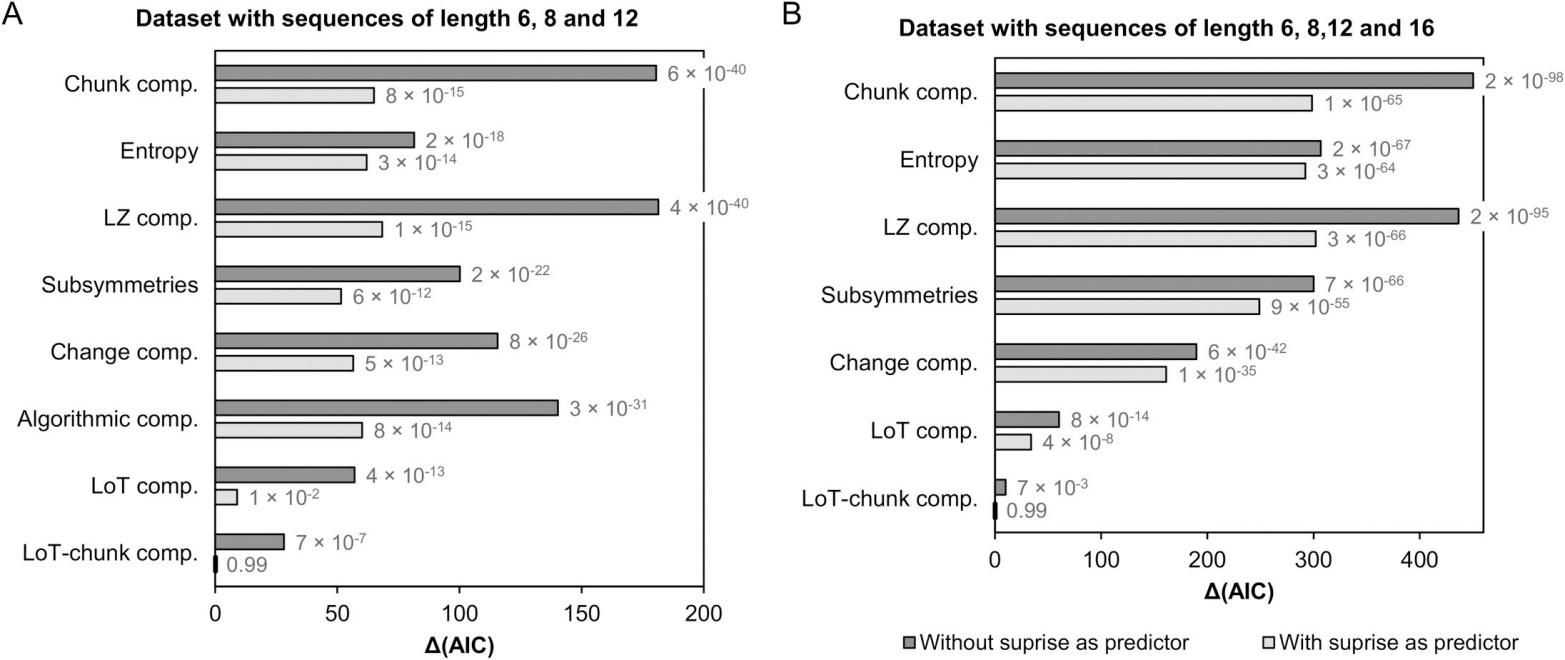
Δ(AIC) for the sixteen mixed models tested using the dataset including the task performance (LISAS) for sequences of length 6, 8 and 12 (A), and for the twelve different mixed models tested using the dataset with sequences of length 6, 8, 12 and 16 (B). The fixed effect of interest is indicated along the vertical axis (all models included participants as a random effect and could include surprise as a covariate—light gray bars). Akaike weight for each model is also reported. The model with lower AIC (Δ(AIC) = 0) is indicated by short dark vertical line on the vertical axis.

Sixteen different mixed models were fitted using datasets with sequences of length 6, 8 and 12. As illustrated in Fig 8A, model fit, as indexed by the Δ(AIC) value, always improved when the surprise associated to the deviants was included in the model. This finding confirms that the effect due to transition probabilities needs to be taken into account when assessing responses to deviants in the violation detection paradigm. The improvement in model fit was smallest for the model with entropy. This effect was expected since entropy and surprise are two tightly related information measures (Shannon entropy is the average of Shannon surprise).

When considering either only single-predictor models (i.e. without the surprise covariate) or two-predictors models (i.e. with surprise), the two best models were the ones with our modified version of LoT complexity (i.e. LoT-chunk, with the “no-splitting” chunks constraint) followed by the one with original LoT complexity (see Table 2). In order to test whether the differences in the raw AIC values were relevant, we computed the Akaike weights for this set of 14 models. Akaike weights can be interpreted as the probability that a given model is the best model of the set [106]. Akaike weight was .99 for the LoT-chunk complexity (+ surprise) model, .01 for the LoT complexity (+ surprise) model, and below .01 for all other models (see Table 2 and Fig 8A).

**Table 2:** Model comparisons for the each of the two datasets.

	*Dataset with sequences of length 6*, *8 and 12*				*Dataset with sequences of length 6*, *8*, *12 and 16*			
*Model fixed effect(s)*	Log-lik.	Δ(AIC)	Δ(BIC)	w(AIC)	Log-lik.	Δ(AIC)	Δ(BIC)	w(AIC)
*LoT comp*.	-14886	57	51	4.0 × 10^−13^	-16653	60	55	7.8 × 10^−14^
*LoT comp*. *+ Surp*.	-14861	9	9	1.1 × 10^−2^	-16639	34	34	4.1 × 10^−8^
*LoT-chunk comp*.	-14872	28	23	7.4 × 10^−7^	-16628	10	4	6.8 × 10^−3^
*LoT-chunk comp*. *+ Surp*.	-14857	0	0	0.99	-16622	0	0	0.99
*Chunk comp*.	-14948	180	175	6.4 × 10^−40^	-16848	450	445	1.6 × 10^−98^
*Chunk comp*. *+ Surp*.	-14889	65	65	7.5 × 10^−15^	-16771	299	299	1.4 × 10^−65^
*Entropy*	-14899	81	76	2.0 × 10^−18^	-16776	307	301	2.3 × 10^−67^
*Entropy + Surp*.	-14888	62	62	3.4 × 10^−14^	-16768	292	292	3.3 × 10^−64^
*LZ comp*.	-14948	181	176	3.9 × 10^−40^	-16841	436	431	1.6 × 10^−95^
*LZ comp*. *+ Surp*.	-14891	68	68	1.4 × 10^−15^	-16773	302	302	2.6 × 10^−66^
*Subsymmetries*	-14908	100	94	1.2 × 10^−22^	-16773	300	294	8.8 × 10^−55^
*Subsymmetries + Surp*.	-14883	52	52	6.2 × 10^−12^	-16746	249	249	1.3 × 10^−17^
*Change comp*.	-14916	116	110	7.6 × 10^−26^	-16718	190	184	6.2 × 10^−42^
*Change comp*. *+ Surp*.	-14885	57	57	5.3 × 10^−13^	-16703	161	161	1.0 × 10^−35^
*Algorithmic comp*.	-14928	140	135	3.4 × 10^−31^	*N*.*A*.			
*Algorithmic comp*. *+ Surp*.	-14887	60	60	8.4 × 10^−14^	*N*.*A*.			

*Note*. All models included participants as a random effect, and either one or two fixed effect(s) (i.e. “+ Surp.”: with additional surprise fixed effect). Log-lik. = log of the maximum likelihood for the model. Δ(AIC) = AIC difference with the model with the lowest AIC value (where AIC is the Akaike Information Criterion). Δ(BIC) = BIC difference with the model with the lowest BIC value (where BIC is the Bayesian Information Criterion). w(AIC) = Akaike weight.

Although correlations between performance and LoT complexity in experiments 2, 3 and 4 (lengths 6, 8 and 12) were small compared to experiment 1 (length 16), LoT complexity again appears as the best predictor of performance in the violation detection task with sequences of length ≤ 12. Notably, the constraint of excluding, for each pattern, the expressions that resulted in the splitting of a chunk (before the selection of shortest expression) improved the fit of behavioral data. This observation suggests that participants did not always find the best way of coding some patterns (best in the sense of the language of thought considered here) because of a propensity to perform an initial chunking solely based on consecutive runs of identical items.

**Fig 9:**
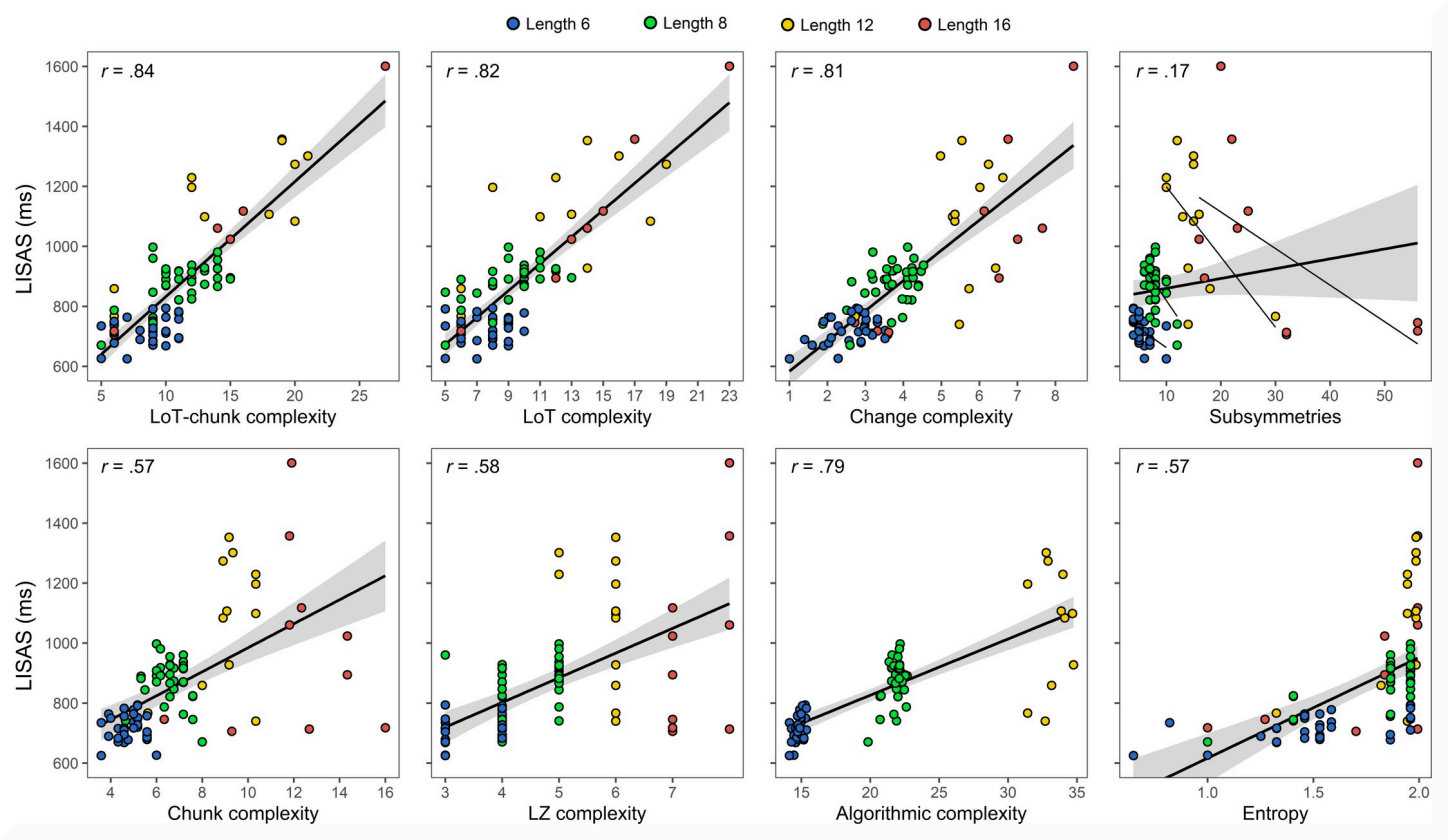
Linear regressions of average performance per sequence (LISAS, in ms) with eight different predictors of interest when combining data from experiments with auditory sequences of 4 different lengths. Each marker corresponds to one sequence. Sequences of different lengths are indicated by different markers only for illustration purposes (the length factor was not taken into account when computing the correlation coefficient, *r*). 16-items long sequences (as well as one 12-items sequence) could not be included in the regression with algorithmic complexity. Regressions lines for each sequence length were added in the subsymmetries plot, in order to illustrate the fact that negative correlations were observed when each length was considered separately. Note that the average performance data presented here does not take into account the effects of surprise, inter-subject, or inter-experiment variability.

The next best model was the one with the “number of subsymmetries” predictor (and including the surprise covariate), suggesting that it also provides a good measure of the psychological complexity of patterns. However, while this appeared true here using statistical models partially controlling for sequence length (i.e. by including participant index as a random factor, since each participant performed the task with only one given sequence length), this measure appears inappropriate to predict complexity across different lengths. Indeed, when we computed the Pearson correlation of average LISAS per sequence for the pooled dataset (sequences of length 6, 8 and 12), we obtained a *positive* correlation value of .39. Such positive correlation is in conflict with the presupposition that patterns containing more symmetries should be simpler. This is explained by the fact that the number of subsymmetries tends to increase with sequence length. These correlations were actually negative when each length was considered independently (*r* = –.44 for length 6; *r* = –.54 for length 8; and *r* = –.58 for length 12). This is illustrated in Fig 9, where the average LISAS for each sequence is presented in relation to each complexity measure (see also S9 and S10 Figs for the equivalent with reaction times and miss rates). To summarize, although this measure is quite good in predicting the complexity of sequences for given length, it fails in predicting the variations in complexity across sequence lengths.

Another similar limitation applies to algorithmic complexity, where the correlation observed across lengths (*r* = .79) is mostly explained by the fact that complexity values present excessive discontinuities with length: algorithmic complexity ranges roughly between 14 and 16 for length 6; between 19 and 23 for length 8; and between 31 and 35 for length 12 (see Fig 9). Such a massive increase in complexity with length is not consistent with behavior. Again, LoT complexity provides a better correlation with the present behavioral data across a large range of sequence lengths, because it correctly predicts that, for instance, some 6-items long sequences can be more complex than some 12-items ones (e.g. ABAAAB, LoT complexity = 10, means LISAS = 778 ms; AAAAAABBBBBB, LoT complexity = 6, mean LISAS = 766 ms).

### Dataset with sequences of length 6, 8, 12 and 16

Fourteen different mixed models (with participants as a random effect) were here fitted, using the same dataset as before to which was added data from 11 sequences for which algorithmic complexity value was not available (thus now with sequences of length 6, 8, 12 and 16). The same predictors as above were used, with the exception of algorithmic complexity. Here again, as illustrated in Fig 8B, goodness of fit systematically increased when surprise was included. LoT-chunk complexity and LoT complexity (with or without surprise as a covariate) were again the best predictors of performance (see Table 2). As opposed to the previous set of analyses in which the data from experiment 1 (length 16) was not included, the model with change complexity performed clearly better than the one with the number of subsymmetries. The long sequences used in experiment 1 indeed presented important differences in their number of subsymmetries (e.g. 56 for (AB)^8^ vs. 32 for (A^4^B^4^)^2^), which were clearly not predictive of performance. Consequently, and as stated earlier, the number of subsymmetries does not appear as a good predictor of task performance across different sequence lengths. Change complexity also appeared as a much better predictor when performing a simple linear regression on average LISAS per sequence (see Fig 9), resulting in an *r* = .81, which is close to the one obtained with LoT complexity (*r* = .82). It indicates that change complexity can also be a good measure of the psychological complexity of a sequence regardless of its length. It must however be noted that, contrary to mixed models, these linear regressions using data averaged over participants did not control for the variance accounted for by surprise, or due to inter-subject variability. Important variations in the correlation with complexity (especially for experiments with shorter sequences) were indeed observed across participants. When computed at the level of individual participants, the correlation with LoT complexity appeared on average stronger (mean *r* = .31, *SD* = .32) than the one with change complexity (mean *r* = .23, *SD* = .30; t(112) = 3.54, p < .001).

With both datasets, two measures performed poorly, LZ complexity and chunk complexity. Contrary to our language, the LZ algorithm has the advantage to be able to quickly “parse” any sequence of any number of different characters, by building for each sequence its own vocabulary of substrings. Its adequacy to human behavior, however, appears limited since, when scanning the sequence from one item to the next, it does not necessarily take into consideration runs of repeated items (AAA can be described with two substrings, A and AA) and fails to capture repeating patterns. This deficiency is especially striking for a low LoT complexity sequence such as (A^2^B^2^)^4^ (i.e. AABBAABB…), where 8 substrings are present in the vocabulary at the end of scanning (the first four substrings encountered by the algorithm are A, AB, B, AA). This gives this sequence the lower level of LZ compressibility among those tested, which is clearly not predictive of performance.

Similarly, “chunk complexity”, like other methods solely based on quantifying chunks (number of chunks, chunks length, or a combination of both), is strongly dependent on how chunks are defined. Here, since chunks are defined as runs of identical items, the complexity of sequences containing alternations tends to be overestimated (e.g. ABABABAB has 8 chunks). Assessing complexity based on chunks therefore requires first building a model that defines what chunks are for the sequence processing cognitive system, which is not trivial. Another limitation of this measure is an excessive sensitivity to sequence length. In the absence of any recursive compression, complexity increases linearly with the number of chunks. Allowing compression based on consecutive repetitions of chunks (chunks of chunks), as in the LoT model proposed here, appears to be a better strategy for predicting the subjective complexity of sequences. Note that, notwithstanding the aforementioned concerns, change complexity captures relatively well the complexity variations due to both structure and length (Fig 9). This may be due to the fact that change complexity is computed within substrings of all possible lengths, which is another way to capture regularities at multiple hierarchical levels.

Unlike several other experimenters, we used an objective deviant detection task to index the psychological complexity of auditory and visual patterns. However, we also collected subjective complexity rating in experiment 1 and 2 (with respectively 10 and 12 sequences), which we therefore also fitted to the various models. In experiment 1, the results were quite consistent in favoring LoT complexity (r = .99 for deviant detection, r = .94 for subjective complexity rating), LoT-chunk complexity (r = .99 and r = .93) and change complexity (r = .89 and r = .95), while entropy (r = .58 and r = .70) and subsymmetries (r = -.63 and r = -.82) led to lower and less consistent results. Similarly in experiment 2, the correlations were good with LoT complexity (r = .60 for deviant detection, r = .61 for subjective complexity rating) and Lot chunk complexity (r = .72 and r = .71), but surprisingly, other measures now provided equally good or even better fits: change complexity (r = .28 vs. r = .65) and especially entropy (r = .43 vs. r = .78) and subsymmetries (r = -.44 vs. r = -.85). Although these results must be treated with caution since they come from a relatively small number of sequences and trials, they may indicate that the internal code for sequences is not entirely accessible to introspection and that, therefore, subjective ratings do not always faithfully reflect the subjects’ objective memory abilities.

**Fig 10:**
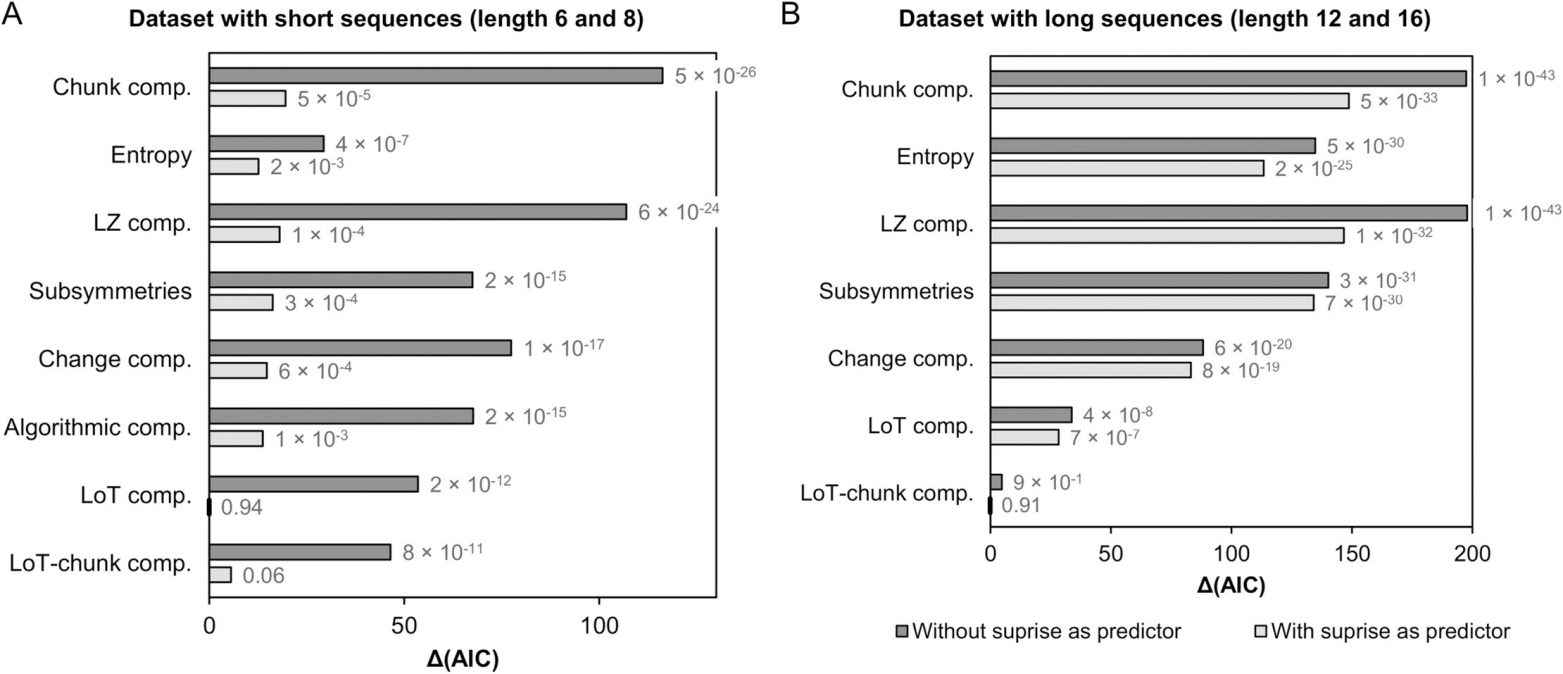
Complementary mixed model comparison with two pooled dataset. A) Δ(AIC) for the sixteen mixed models tested using a dataset including the task performance (LISAS) for sequences of length 6 and 8 (66 sequences) (A), and for the twelve different mixed models tested using the dataset with sequences of length 12 and 16 (22 sequences) (B). The fixed effect of interest is indicated along the vertical axis (all models included participants as a random effect and could include surprise as a covariate—light gray bars). Akaike weight for each model is also reported.

It could be argued that the above results may be biased because we started with a preconceived language-of-thought and selected sequences whose structures were well-captured by that language (as well as some sequences that were maximally irregular according to that language). Although such a bias cannot be definitively ruled out, there are several arguments against it. First, this potential problem does not apply to experiments 3 and 4, where we tested *all* appropriate sequences of length 6 and 8, in an unbiased manner (the only restriction for sequences of length 8 was to have the same number of As and Bs). An additional model comparison analysis, restricted to those sequences, revealed that our complexity metrics remained the best predictors (see Fig 10A). Very similar results were obtained when including only the set of length-8 sequences, which appears to be the minimum length at which compression effects have been observed (see S11 Fig). Second, for longer sequences, exhaustive sampling would have been impossible, and random sampling would have been equally inappropriate. This is because for any reasonable notion of complexity, only a very small number of sequences achieve a low complexity, while the vast majority of randomly selected sequences achieve a high level of complexity [43,107]. Thus, some selection of sequences was required in experiments 1 and 2, with length 16 and length 12 respectively. The graphs in Fig 9 nevertheless indicate that our selection was not particularly biased, inasmuch as the values of, for instance, change complexity or entropy spanned across a broad range and therefore would have permitted those variables to win over LoT complexity in our regressions, if they had been the best predictors. In spite of this relative “theory neutrality” of our length-12 and length-16 sequences, we again found an advantage in favor of the LoT and LoT-chunk predictors (Fig 10B) when model comparison was restricted to them. Furthermore, even when restricting the analysis to a subsample of thirteen length-12 and length-16 sequences for which change complexity was approximately constant (between 5 and 7), we still found a correlation of performance with LoT-Chunk complexity (r = 0.70, p = 0.007) and a marginal one with LoT complexity (r = 0.54, p = 0.057), while the correlation with change complexity was naturally no longer present, r = .23, p = .45). Finally, note that although our research was indeed initially predicated on the idea that LoT complexity would be the best predictor of human behavior, the data was unbiased enough to lead to a different conclusion, namely that Lot-chunk complexity was a superior predictor. Nevertheless, we acknowledge that our experiments were not specifically designed to arbitrate between different models of sequence complexity with respect to their capacity to predict behavior (especially regarding the selection of the longer sequences). The set of long sequences used here represents only a tiny sample of all possible combinations and structures and, in spite of the above arguments, it cannot be definitely excluded that models other than ours would be better at capturing psychological complexity if a different set was used. Future studies could focus on the isolation of sequences for which different models make opposite predictions. Such a situation, although relatively rare (because different complexity metrics tend to correlate with each other) may exist within the large number of available sequences and should provide more definitive data.

## General discussion

The main goal of this series of experiments was to evaluate the mental representation of binary sequences and to test the adequacy of a formal language of thought previously proposed to account for geometrical sequences [58]. Similar models were proposed in the past [e.g. 33,37,48] but were not submitted to a full experimental validation, particularly in comparison to the most recent approaches of sequence complexity. Moreover, we sought to distinguish the effects related to statistical transition-probability learning, which are unavoidable when dealing with temporal sequences of stimuli, from the putative influence of rule-based encoding. Across five different experiments with sequences of different lengths, in the auditory but also in the visual modality, we found consistent evidence that, a significant part of the variations in sequence encoding performance (as indexed by the capacity to detect sequence violations) was explained by the length of the shortest possible description of the sequence in the proposed formal language (i.e. LoT complexity). This was however not the case for very short sequences (6 items). These results are consistent with the idea that upon hearing or seeing a binary sequence, when the number of items exceeds working memory capacity, subjects compress the sequence into an abstract, language-like mental representation. It is remarkable that a language merely composed of two simple instructions (“same” and “change”) and their recursive embeddings accounts for a large amount of the formation of such a representation. The complexity measure derived from this language was moreover better predictive of the degree of psychological complexity than other sophisticated approaches designed as alternatives to the non-computable Kolmogorov complexity [46,47].

The assumption that the length of the shortest description in the formal language corresponds to perceived sequence complexity was further corroborated by subjective complexity rating (experiments 1 and 2). Moreover, we found that sequence structure was not the only information encoded by participants: surprise levels derived from the statistical estimation of transition probabilities also consistently explained part of the variance in violation detection performance. The effects of surprise and of complexity on responses to violations were found to vary differently depending on sequence length, thus providing new insights on how the human brain makes predictions in temporal sequences.

The predictive power of the LoT was most notable for the longest sequences, in particular for 16 items long sequences (experiment 1; *r* = .98). Indeed, large differences in miss rates were observed between sequences predicted to be the least (A^*n*^B^*n*^ patterns, with LoT complexity = 6) and the most complex (a set of 10 instructions, LoT complexity = 23), suggesting that subjects simply could not learn the latter efficiently (even after eight or more repetitions). An additional prediction of LoT was verified, namely the fact that the four sequences based on the A^*n*^B^*n*^ pattern were associated with a similar performance level, regardless of *n* (= 1, 2, 4, or 8). In the language, this is because the complexity of a repetition is proportional to the log-number of repetitions (rounded up to the nearest integer). For a total number of 16 items, it therefore does not matter if the sequence is decomposed in 2 chunks of 8, 4 chunks of 4, 8 chunks of 2, or 16 chunks of 1: the sum of weights remains unchanged, leading to a LoT complexity of 6 bits in all cases—and indeed, the observed performance remained stable across such a broad variation ranging from huge chunks to pure alternation (see Fig 3).

The correlation of performance with LoT complexity decreased in subsequent experiments using increasingly shorter sequences, until it became almost absent for sequences comprising only six elements. Rather than an indication of an intrinsic limitation of the language for describing very short binary patterns, we believe that a significant part of this effect relates to differences in working memory demands. The number 6 indeed falls within the usual limits for the number of items that can be stored in working memory, which is around 7±2 items when there is no compression [16,29]. Thus, subjects could have solved the violation detection task without compression, purely by storing each 6-items sequence “as is” in working memory. Similarly, 8-items sequences could have been stored as a mere flat series of “chunks”, which are thought to be the units of encoding in working memory [16,85,108,109], without any recursive embedding. All in all, an increasingly greater need to rely on compression would explain why the predictive power of LoT complexity increases with sequence length.

Although the definition of working memory chunks as “a collection of elements having strong associations with one another” [25,110] is too vague to be rigorously tested using the present data, it is easy to imagine that both conceptions can lead to similar predictions (sequences composed of a small number of small chunks also have a short description in our language). Note however that, when considering all tested sequences, LoT complexity outperformed the “chunk complexity” predictor, for which chunks are defined using consecutive repetitions of the same item. In fact, a crucial feature of our theory lies in going beyond a simple concatenation of chunks and forming recursively embedded or nested representations, that is the ability to represent “chunks of chunks” or “repetitions of repetitions”. Indeed, the construction of recursively nested structured has been proposed as a core human ability, which sets us apart from other primates [4,6,7,111]. Our results support the idea that the inclusion of such a feature is essential to explain human behavior when working memory capacity is exceeded and compression is most beneficial.

The fact that we reached such a conclusion using the simplest type of temporal sequences (binary sequences) and a simple deviant detection task (rather than the more demanding recall, completion or production tasks using in the previous literature) is consistent with Fitch’s “dendrophilia hypothesis” [8] which states that “humans have a multi-domain capacity and proclivity to infer tree structures from strings” even in the simplest cases. The present work provides a foundation for future experiments in non-human primates, which would allow us to test the second aspect of this hypothesis, namely that this capacity for building recursive tree structures is only available to humans [4,6,8]. In non-human primates, we postulate that a simpler language will suffice to account for sequence coding.

Numerous other frameworks for the estimation of pattern complexity have been proposed in the past, such as change complexity [47], algorithmic complexity [44–46], subsymmetries [93] or entropy [see also 35,94–96,112]. These models are often based on quantitative aspects of information, such as the length, the number of transitions or runs, the probability of those transitions, the number of symmetries, or the number of changes. Although they all show some level of success in predicting behavior, they fail to capture recursive nesting, which as noted above seems to be an essential factor in human cognition [4,6]. The same limitation applies to the Lempel-Ziv data compression algorithm, which compresses sequences by storing in memory a set of unique substrings that can occur at different locations in a sequence. Although it may seem psychologically relevant, this specific algorithm is unable to consider relationships between substrings mediated by an abstract, higher-level operation of repetition or change, as a LoT model does. In addition, this algorithm does not take advantage of contiguous repetitions. Conversely, the notion of repetition with variations is central to the success of our language. Others have also proposed that humans possess a “repetition detector”, as they are much better to learn repetition-based grammars than other forms of simple grammars [113]. Such increased sensitivity for repetitions (compared to alternations) also follows from the simple assumption that humans track transition probabilities at a local scale [21]. Repetition detection may already be present at birth, which suggests that it may be an innate neurocognitive function, perhaps essential for language acquisition [114]. It may therefore not be surprising that nested repetitions with variations suffices to account for the human memory for sequences, and that models that do not incorporate this struggle to replicate human behavior.

Following others in the domain of concept learning [e.g. 52,56], the approach adopted here assumes that binary sequences are encoded using a specific cognitive system that manipulates abstract, symbolic representations—a language of thought with recursive calls to a limited number of primitive operations. Thus, the present proposal does not merely provide a numerical value for complexity, but also parse trees and precise internal formats of representations, both of which could possibly be tested in future behavioral or brain-imaging experiments.

Although the current study is based on the use of a “fixed” language, with predetermined rules and associated weights, some evidence suggests that a better description of human behavior can be achieved by incorporating a probabilistic component to the modeling attempt. This approach, advocated by Piantadosi & Jacobs [53] under the term *probabilistic language of thought* (pLOT), consists in using Bayesian probabilistic inference to estimate the likelihood of the existence of some set of rules (a proposed formal language), given the observed data. It has been shown to be especially efficient in modeling concept learning, for instance by replicating the patterns of errors throughout learning [50,52,56]. This approach was also adopted to investigate how humans assess randomness in their environment. Human biases in subjective randomness judgments [e.g. 115,116] could be explained by assuming that the representation of randomness results from a statistical inference about the processes that generated the sequence [21], i.e. an estimation of the probability that a given regular process produced it [117]. A good fit to human behavior was obtained without using the full power of Turing machines, but only finite-state automata with a stack, which are able to recognize repetitions, alternations or symmetries [18,117]. Thus, despite fundamental differences (notably, deterministic versus probabilistic languages), the pLOT theory shares with our approach the need to consider similar types of primitive operations. Given the strong links between subjective randomness and complexity, we can reasonably expect that our formal language may also predict whether a pattern is perceived as random or not—a possibility which remains to be tested in future work.

Beside the learning of conceptual knowledge and work on subjective randomness, a pLOT approach was also used to model the learning of spatial sequences: to study the crossmodal transfer of sequence knowledge [92], and to investigate the adequacy of the language of geometry [57]. Indeed, by using the behavioral data from the octagon task of Amalric et al. [58], Romano et al. [57] showed that the primitives included in the language of geometry were all required in order to best account for human behavior. In spite of its successes, a number of questions and potential limitations of the LoT approach remain. First, the construction of our formal language implied methodological choices that could be considered as arbitrary or at least requiring more experimental validation. The primitive instructions included in our formal language were chosen for their alleged simplicity and because they suffice to represent any binary sequence. Other primitives could be tested (e.g. counting and a system of arithmetic; or temporal inversion or “mirroring”, see 10). Furthermore, modifications of the weights associated with each instruction or their number of repetitions may lead to different estimates of complexity. Finding the correct language for a given population is crucial, especially in the context of the debate on the uniqueness of human sequence processing skills, and specific statistical methodologies need to be developed for this purpose. As mentioned earlier, the pLOT approach which, using Bayesian inference, allows to find the most likely concepts and rules from a grammatically structured hypothesis space containing several candidates, appears to be a very promising approach for that purpose [50,53,57]. Nevertheless, we also found that some of the minimal expressions produced by this language did not fit well with the way participants represent some sequences. The addition of the constraint that the minimal parse tree should respect the chunks or runs of consecutive repetitions, and never split any such chunk, was found to lead to a noticeable improvement in model fit. We speculate that this finding reflects the way participants build their internal representation of sequences: since the space of possible programs is immense, they would restrict the search to only those programs that, at the lowest level, generate the observed consecutive runs in the sequence. The perceptual dominance of the runs could act as a bottleneck, an initial grouping that would then restrict the sequence parsing process (as is sometimes assumed in some complexity estimation models; e.g. [96]). A better characterization of this parsing process during sequence learning could help address the current limitations of our language.

Another limitation is that, although we argued that the capacity to represent sequences using hierarchically embedded or nested descriptions is an essential feature of human behavior [4], about half of the minimal expressions for the sequences that we used included only two hierarchical levels (a single level of embedding; the average hierarchical depth was 2.5). Only a few sequences such as AABBABABAABBABA explicitly required repetitions of repetitions of repetitions. Although our model correctly predicted their subjective and objective complexity (see Fig 3), and although embedding is an effective compression process, more research is needed to probe whether human participants always consider such deep levels of embedding as beneficial in the processing of short sequences. Increasing the hierarchical depth may imply an additional processing cost, making it useful only in specific situations (e.g. for more demanding learning tasks or with long sequences).

Finally, our approach assumes that the mental compression of sequences does not necessarily occur at the level of the sensory items (i.e. grouping contiguous identical elements) but at the more abstract level of the relationships between items. Besides its success in predicting the psychological complexity of sequences of tones, one argument in favor of such an abstract symbolic representation is that it fitted equally well the complexity of visual sequences. However, it could be proposed that the mental encoding of temporal sequence does not involve any amodal, domain-general processing mechanisms, but rather two similarly organized modality-specific systems, or even a single modality-specific cognitive system dedicated to auditory processing; visual sequences would then be converted into an auditory representation prior to compression. Indeed, we observed a lower performance and slower responses in the visual compared to the auditory modality, a difference which has been postulated to reflect a dominance of the auditory system for the encoding of temporal information [90,118,119]. One potential strategy for performing the task of experiment 5 with visual stimuli could have been a subvocal naming of the items, and a maintenance in working memory using the phonological loop [30,120]. Further investigation is required to resolve these points, perhaps by relying on other sensory modalities, by testing transfer across modalities, or by using brain-imaging to determine the sensory versus higher-level nature of the brain mechanisms at play. We merely note here that activation of supra-modal prefrontal cortices has been reported during sequence processing [19,60]; that the existence of an automatic visual-to-auditory conversion in sequence processing has been challenged [121]; and that the existence of an abstract representation of sequences as proposed here, allowing a transfer of knowledge across modalities, is already supported by some behavioral data [see 92].

The violation detection task used in the present study implied the learning of a specific and deterministic sequence in each block, which was repeated multiple times with predictable timings. Our results, however, indicate that the statistical properties of the original sequence were also computed in parallel to the compression process and used for prediction, since, for a given sequence, performance varied according to the level of surprise, i.e. the negative log transition probability of the deviant sound in the context of the current sequence. For equal complexity, we observed a higher accuracy and faster response times for deviants that induced less frequent transitions. The observation that transition probability affects behavior even within a deterministic sequence [see also 84], as opposed to the stochastic sequences that were used in previous studies of statistical learning [e.g. 19–21,78,79,122], suggests that the learning of transition probabilities between items may occur automatically and in parallel to compression in working memory. This is compatible with the large amount of evidence showing that the brain encodes statistical regularities in sensory inputs in an implicit and unconscious manner [72,123–126]. Since the effect of surprise occurred over and above any effect of sequence complexity, it also suggests that this statistical learning system is distinct from the more strategic system based on the learning of the deterministic sequence structure. Again, this is compatible with prior brain imaging results on the local-global paradigm, which indicate that the mismatch negativity (MMN), sensitive to local transition probability, can be dissociated from the P3b response associated with the acquisition of the global sequence [66,67,71].

When pooling datasets from experiments with different sequence lengths, the linear mixed models with surprise and complexity as predictors fitted the data better than models including one predictor alone, indicating that those two predictors captured distinct aspects of the data. However, one may note that the size of the surprise effect varied across experiments. Surprise and complexity showed opposite patterns, with a stronger effect of complexity for longer sequences than shorter ones and, conversely, a strong effect of surprise only with the shortest sequences. Given the evidence that we just cited, showing that transition probabilities are constantly being computed unconsciously, the most likely interpretation is probably that task difficulty increased with sequence length and resulted in longer response times, thus masking the contribution of statistical learning. To test this idea, future work should use event-related potentials such as the MMN, which may provide a more sensitive measure of transition-probability learning.

Finally, we found a complexity effect even when subjects responded to “super-deviants” items, i.e. outlier sounds that could be detected without any knowledge of the sequence because their identity itself was novel. We suggest two putative interpretations of this unexpected effect. First, it could be due to the increased attentional load associated with more complex sequences. Essentially, participants would be placed in a dual-task situation of having to attend to two things are once: the complex sequence and the occasional deviants. In support of this idea, increased attentional load has indeed been found associated to sequence learning impairment in dual-task experiments [see 127]. A second interpretation, within the predictive coding framework, is that deviance detection, even for extremely salient deviants, is easier for predictable than for unpredictable stimuli. Accordingly, Southwell and Chait [128] found larger brain responses evoked by deviant stimuli within a regular sequence than within a random sequence of tones. The authors propose that it could reflect a difference in the *precision* or predictability associated with the flow of sensory information. Indeed, in addition to the prediction regarding the content of incoming stimuli (manifested by prediction error signals), recent versions of predictive coding theories also formalize the concept of precision, which corresponds to the reliability of the prediction [79,129–132]. Precision would manifest itself as a gain modulation of the relevant neural units (which is tightly related to attention), with increased precision leading to an increasing sensitivity to the predicted stimuli. This theory can explain the increased and sustained neuronal responses observed in a highly predictable context [126,128,129,133]. The present complexity effect observed for super-deviants may thus indicate that responses to completely unexpected events were modulated by the degree of predictability of the pattern, which itself depends upon the complexity of the pattern. A precision-weighting mechanism would thus explain why greater complexity leads to slower response times to any kind of violations in our violation detection task. Overall, the distinct contributions of surprise and complexity underline the joint contributions of statistical versus rule-based information in temporal sequence processing.

## Conclusion

Our study provides a first demonstration that, even after accounting for statistical transition probability learning, responses to sequence violations can be used to uncover the properties of the abstract mental language used by individuals to encode sequential patterns. The present proposal, which takes the form of a psychologically plausible formal language composed of a restricted set of simple rules (conforming to a simplicity principle and especially relying on the human ability to detect repetitions), proved to be more effective than alternative approaches in modeling the human memory for simple sequences. The observed relationship between sequence complexity and performance in the detection of violations is consistent with the idea that the brain acts as a compressor of incoming information that captures regularities and uses them to predict the remainder of the sequence. The present non-verbal passive paradigm paves the way to future neurophysiological recording studies that would probe the similarities and differences between humans and other species [13] or test the abilities of preverbal infants [69]. A fundamental question for future research is whether the same formal language can explain sequence processing in other primate species, or if such a language is unique to humans [6].

## Materials and methods

### Ethics statement

Experiments were approved by the regional ethical committee (Comité d’Ethique pour la Recherche, CER, de l’Université Paris Saclay), participants gave written consent to participate and were paid for their participation.

### Participants

Twenty-eight healthy volunteers (*M*_age_ = 24.3, *SD* = 3.2, 16 women) participated in experiment 1, twenty in experiment 2 (*M*_age_ = 26.5, *SD* = 9.5, 15 women), thirty-two in experiment 3 (*M*_age_ = 27.4, *SD* = 5.3, 21 women), twenty-three in experiment 4 (*M*_age_ = 23.4, *SD* = 4.5, 18 women) and eighteen in experiment 5 (*M*_age_ = 25.5, *SD* = 5.7, 15 women). They all gave written consent to participate and were paid for their participation. In experiment 1, all participants performed the subjective complexity rating task but, due to time constraints, seven of them performed only 6 out of the 10 independent short sessions of deviance detection.

### Stimuli

Auditory binary sequences used in all five experiments were composed of an alternation of low pitch and high pitch tones. Each stimulus was a complex tone synthesized with the superimposition of four sine waves. Sound frequencies were chosen to correspond to musical notes: 494, 740, 988 and 1480Hz (i.e. B, F#, B, F#) for the low pitch tone, and 622, 932, 1245 and 1865Hz (i.e. D#, Bb, D#, Bb) for the high pitch tone. The two complex tones were randomly assigned to items A and B for each experimental session. Thus, stimulus attribution changed from one sequence to the next and from one participant to the next but was kept constant for a given sequence in a given participant. In addition, one lower pitch tone (415, 622, 831 and 1245Hz) and one higher pitch tone (740, 1109, 1480 and 2217Hz) were synthesized, to be used as easy-to-detect super-deviant (or C) stimuli in experiments 1 and 2. All tones were 50 ms long, with 5 ms initial and final ramp. Inter-stimulus interval (ISI) was 200 ms.

Ten 16-items long sequential patterns were chosen for experiment 1 (see Fig 2), which were all composed of the same number of items (8 As, 8 Bs), giving a total sequence duration of 3800 ms. The first four sequential patterns, of lowest complexity, followed the simple algebraic pattern (A^*n*^B^*n*^)^*x*^: (AB)^8^, (A^2^B^2^)^4^, (A^4^B^4^)^2^ and A^8^B^8^. The period of these sequences differed (2, 4, 8 and 16 tones), but the complexity was identical (LoT complexity = 6). Although the shortest description formula did not necessarily conform to our intuitive A^*n*^B^*n*^ notation (i.e., [[+0]^16<b>], [[[+0]^2]^8<b>], [[[+0]^4]^4<b>], and [[[+0]^8]^2<b>], respectively), they are indeed all represented with a formula containing 2 instructions and 2 digits. The other 6 sequences had LoT complexity values ranging from 12 to 23. Half of them were periodic (period of 8).

In experiment 2, twelve 12-items long different sequential patterns, each composed of 6 As and 6 Bs were presented to each participant (Fig 4). Sequence duration was 2800 ms. In experiment 3, thirty-five 8-items long different sequential patterns, each composed of 4 As and 4 Bs, were used (see S3 Fig), i.e. all possible 8-element-long binary combinations that contained the same number of As and Bs. Sequence duration was 1800 ms. In experiment 4, thirty-two 6-items long different sequential patterns were used (see S4 Fig), representing all 2^5^ types of 6-element sequences (given that the labelling of As and Bs is arbitrary, sequences such ABABAB and BABABA were considered identical). Note that, in this case, the proportion of As vs. Bs varied across sequences. Sequence duration was 1300 ms. In experiment 5, fifteen 8-items long sequential patterns were used (see S7 Fig). All were previously used in experiment 2. They were selected based on their LoT complexity, in order to preserve a large and homogenous distribution of complexity values. The same sequences were presented to participants in auditory and visual forms (in different blocks). Auditory sequences were composed of the same two complex tones as in the previous experiments. Visual sequences were composed of two colored Gabor patches presented in the center of the screen (a red Gabor patch with 45° orientation, and a green patch with 135° orientation). Stimulus duration was 200 ms with 200 ms inter-stimulus interval in both modalities. Sequence duration was 3000 ms.

### Procedure

Participants were seated in front of a computer in a quiet room and were wearing headphones. Stimuli were delivered using the Psychophysics Toolbox 3 [134,135] running on Matlab R2016a (Mathworks Inc., Natick, MA, USA). Before starting the experiment, participants listened to a sample of stimuli (different sequences from the ones used in the main experiment) and the sound volume was adjusted if necessary.

In the first part of experiment 1, participants performed the complexity rating task. They were asked to judge each sequence on a scale going from “1: very simple” to “9: very complex”, by pressing the corresponding key on the keyboard following sequence presentation. They were informed that “each sequence contains two different beeps, presented according to a more or less complex order” and listened two examples, presented as a “rather simple” (AABBAABBAABBAABB), and as “rather complex” (ABAAABABAABBBABB). A response was requested at each trial. Each of the ten sequences was presented three times, in a pseudo-random order (30 trials). The low-pitch and high-pitch tone were randomly assigned to either A and B or to B and A at each presentation.

In the second part of experiment 1, the violation detection task, each of the ten sequences was tested in a different short session of approximately 4 min (Fig 1B). Order of sessions was randomized for each participant. Each session comprised three blocks separated by pauses and in which the sequence (3800 ms long) was repeatedly presented with a 600 ms inter-trial duration. In the first block, the habituation block, the unaltered sequence was presented eight times. Participants were asked to listen to the stimuli and try to remember the sequence. In the two following blocks, the testing blocks, participants were asked to respond whenever they detected that the sequence had been altered (by a deviant tone), by pressing the space key of the keyboard as quickly as possible (without waiting until the end of sequence presentation). Each of the two test blocks comprised 18 sequences, 9 of which contained one deviant tone (among the sixteen tones composing the sequence). Two-thirds of the deviant sequences were produced by replacing a tone A by a tone B, or conversely (“sequence deviant” tones, 12 trials per session). The remaining third were obtained by replacing one tone by a low or high-pitch C sound (“super-deviant” tone, 6 trials per session). Deviant tones could occur at only four, equally probable, positions within the second half of the sequence (positions 9, 11, 13 or 15).

The same procedure and material was used in experiment 2. The complexity rating task was performed first (each of the twelve sequences was presented three times, in a pseudo-random order) followed by the violation detection task. In the latter, each sequence was tested in a different short session of approximately 3 min (habituation block of 8 trials, two test blocks of 18 trials each), followed by a pause. Each sequence lasted 2800 ms and was followed by a 1000 ms intertrial blank. Order of blocks was randomized for each participant. Half of the trials in tests block contained one deviant tone (at positions 7, 8, 9, 10, 11, or 12): 2/3 of “sequence deviants”, 1/3 of “super-deviants”. Participants were asked to press the button, as quickly as possible, as soon as they detected that the sequence had been altered.

The same procedure and material were used in experiment 3 and 4 (except that there was no complexity rating task). Each sequence was however tested in a single block of 35 trials (auditory sequence of 1800 or 1300 ms and inter-trial duration of 1000 ms). Alterations of the sequence occur on 1/3 of the trials, starting from the 9^th^ trial (i.e. the habituation phase comprised 8 repetitions). Deviant tones (sounds A replaced by B or conversely—there were no super-deviants in these experiments) were positioned in the second half of the sequence (four or three equiprobable positions). As before, participants were asked to detect if the sequence had been altered by pressing a button as quickly as possible.

In experiment 5, the same procedure and material were used in the auditory blocks. Participants were instructed to fixate the center of the screen in the visual blocks. Each sequence was tested in a short block of approximately 2.5 min., followed by a pause. Since each sequence was presented twice (i.e. in the visual and in the auditory form), the experiment was divided in two sessions of fourteen blocks, separated by a longer pause. Each pattern appeared once in a given session, which comprised equal numbers of auditory and visual blocks. Order of blocks within each session was randomized for each participant. Each block comprised 35 trials (sequence of 3000 ms and inter-trial duration of 1000 ms). The habituation phase contained at least eight trials, alterations of the sequence occur on 1/3 of the remaining trials (i.e. 9 deviant trials). As before, deviant items only appeared within the second half of the sequence (positions 5, 6, 7 or 8). Participants were asked to press the button, as quickly as possible, as soon as they detected a deviant in the sequence.

### Data analysis

In experiment 1, the responses collected in the complexity rating task, ranging from 1 to 9, were normalized for each participant using a *z*-score transformation of the raw ratings within each participant. An average complexity rating was computed for each sequence and subject and entered into a mixed effect model with participant as random factor and LoT complexity value as a fixed effect predictor. Here and in following mixed effect analyses, similar results were obtained using classical repeated-measures ANOVAs with participants as the random factor.

For the violation detection task, a button press occurring between 200 and 2500 ms after deviant stimulus onset was considered a hit (i.e. a correct response). An absence of response during this interval was counted as a miss. Such a long response time window was adopted in order to allow for a potential “delayed-response” strategy (some participants seemed to wait until the end of the sequence, although the target appeared in the middle), but long response times were rare (especially following response time trimming procedures, see below). False alarms were collected and analyzed separately (using a simple linear regression analysis with the LoT complexity predictor). Note that participants were not aware of the number of deviant targets, or their occurrence frequency, and could respond at any time. Thus, only the number of false alarms, rather than a ratio depending on the number of trials, was relevant. The Linear Integrated Speed-Accuracy Score (LISAS) [86,87], an integrated measure of response times and error rates, was used as the main indicator of performance (results with response times and miss rates were quite convergent and are provided in Supporting Information). This score was computed for each sequence, each deviant type in each subject, according to the following formula: $={RT}_{c}+MR\times \frac{S_{RT}}{S_{MR}}$, where *RT_c_* refers to the average response time (of correct responses), *MR* to the miss rate, *S_RT_* to participant’s overall *RT* standard deviation and *S_MR_* to the participant’s overall *MR* standard deviation. These scores were computed after removing extreme response times (2.5 standard deviations (*SD*) above or below the median in each condition and subject, 2.0% of data). Participants with excessive average miss rate over the entire session (i.e. 2.5 *SD* above group median), average response time and/or average number of false alarms were excluded (three participants). All data analyzes were performed in R 3.6.0 [136].

We performed statistical analyses using a mixed model in which the dependent variable was the LISAS for each participant and each cell of the design; participants were the random factor, and LoT complexity and deviant type (sequence deviants vs. super-deviant) were fixed factors. To clarify the interactions, we also computed the same mixed effect model after restricting the data to each deviant type. All computations were performed using the lme4 [137] and lmerTest [138] packages. P-values for each factor were obtained using Kenward-Roger approximation for degrees of freedom [139].

Since statistical properties were also expected to play a role in how participants react to deviant stimuli, another predictor, distinct from LoT complexity, was constructed. We used Shannon surprise, defined as the negative log-predictive probability of the stimulus [21,79,81–83], to characterize how unexpected a deviant stimulus would be for an observer that tracks transition probabilities between successive items in of the original sequence (p(A_t_|B_t-1_), p(B_t_|B_t-1_) which is equal to 1-p(A_t_|B_t-1_), p(B_t_|A_t-1_), and p(A_t_|A_t-1_) which is equal to 1-p(B_t_|A_t-1_) t and t-1 denote the current and previous trial respectively); for binary sequences: *p*(A|A) = 1 –*p*(B|A) and *p*(A|B) = 1 –*p*(B|B). Since the sequence was considered to be already fully learned after the habituation phase, we used fixed probabilities were used (rather than probabilities evolving on a trial-by-trial basis, as used for instance by Maheu et al. and Meyniel et al., [20,21,84]). For instance, in the A^8^B^8^ sequence, *p*(A|A) has a probability of 0.875. Thus, the corresponding surprise of getting an A (instead of a B) at the 9^th^ position is low (−log_2_(0.875)≈0.18 bit). In the same sequence, *p*(A|B) = 0 (since B is always followed by another B), and therefore the surprise of getting an A instead of a B at, say, the 11^th^ position, is maximal. To avoid an infinite when computing surprise, probabilities of 0 were padded by a small but non-zero probability of *p* = 0.01, capping the maximum surprise value at around 6.64 bits. To test whether this would affect our conclusions, complementary analyses were also conducted while excluding deviants with such null probability. Note that, contrary to the LoT complexity, which characterizes a sequence as a whole and is thus identical whatever the position of the deviant, surprise varies with deviant position within the sequence (up to four different values in one given block). Analyses comparing the surprise and LoT complexity predictors were performed using the same mixed model as above, including participants as random effects. To compare a pair of nested models, we used likelihood ratio tests (using the Chi square distribution). When more than 2 models were involved, we computed the Akaike information criterion for each model [140]. Note that both methods penalize for model complexity (i.e. the number of predictors included in the regression), which varies depending on whether, or not, LoT complexity was included in addition to Shannon surprise (see above). Super-deviant trials were not included in these analyses.

In addition to those mixed effect statistics, we also report the results of simple regressions and Pearson correlation coefficient *r* between LoT complexity and either subjective complexity ratings or the LISAS for each sequence, after averaging across participants (this is the *r* value reported in the figures). Supplementary Information figures in provide this statistic for RTs and miss rates.

The same analyses were conducted in experiment 2, 3, 4 and 5 with the exceptions that there was no deviant type factor in experiments 3, 4 and 5 (no super-deviants stimuli) and that some analyses included modality as a categorical two-levels predictor (auditory vs. visual) in experiment 5. Extreme response times were removed (using the same procedure as in experiment 1), and represented 1.2% of all RTs in experiment 2, 1.6% of RTs in experiment 3, 1.6% in experiment 4 and 2.4% in experiment 5. One participant was excluded in experiment 2 (average number of false alarms per sequence more than 2.5 *SD* above the group median), one in experiment 3 (average number of false alarms per sequence more than 2.5 *SD* above the group median), one in experiment 4 (average miss rate more than 2.5 *SD* above the group median) and one in experiment 5 (average miss rate and number of false alarms more than 2.5 *SD* above the group median).

Data collected in all five experiments is available in S1 and S2 Data (average performance for each participant and sequence).

## Supporting information

S1 Fig Linear relationship between task performance and LoT complexity in Experiment 1. A) Average response time and B) average miss rate.
(TIF)

S2 Fig Linear relationship between task performance and LoT complexity in Experiment 2. A) Average response time and B) average miss rate.
(TIF)

S3 Fig Sequences used in Experiment 3. (TIF)

S4 Fig Sequences used in Experiment 4. (TIF)

S5 Fig Linear relationship between task performance and LoT complexity in Experiment 3. A) Average response time and B) average miss rate.
(TIF)

S6 Fig Linear relationship between task performance and LoT complexity in Experiment 4. A) Average response time and B) average miss rate. Note: the circled dot highlights the performance for the sequence AAAAAA, which was excluded from some analyses.
(TIF)

S7 Fig Sequences used in Experiment 5. (TIF)

S8 Fig Linear relationship between task performance and LoT complexity in Experiment 5. A) Average response time and B) average miss rate.
(TIF)

S9 Fig Linear regressions of average response time (RT) per sequence (in ms) with eight different predictors of interest (when combining data from experiments with auditory sequences of 4 different lengths). Note: 16-items long sequences (as well as one 12-items sequence) could not be included in the regression with algorithmic complexity.
(TIF)

S10 Fig Linear regressions of average hit rate per sequence (in %) with eight different predictors of interest (when combining data from experiments with auditory sequences of 4 different lengths). (TIF)

S11 Fig Complementary mixed model comparison with length-8 sequences. Δ(AIC) for the sixteen mixed models tested using a dataset including the task performance (LISAS) for sequences of length 8 (35 sequences).The fixed effect of interest is indicated along the vertical axis (all models included participants as a random effect and could include surprise as a covariate—light gray bars). Akaike weight for each model is also reported.
(TIF)

S1 Data Data from the deviant detection tasks of the 5 experiments. (CSV)

S2 Data Data from the complexity rating tasks of experiments 1 and 2. (CSV)
