# Supplementary figures and images for: A theory of memory for binary sequences: Evidence for a mental compression algorithm in humans
Source: PLoS Comput Biol. 2021 Jan 19;17(1):e1008598. doi: 10.1371/journal.pcbi.1008598 (PMC7845997; doi:10.1371/journal.pcbi.1008598)

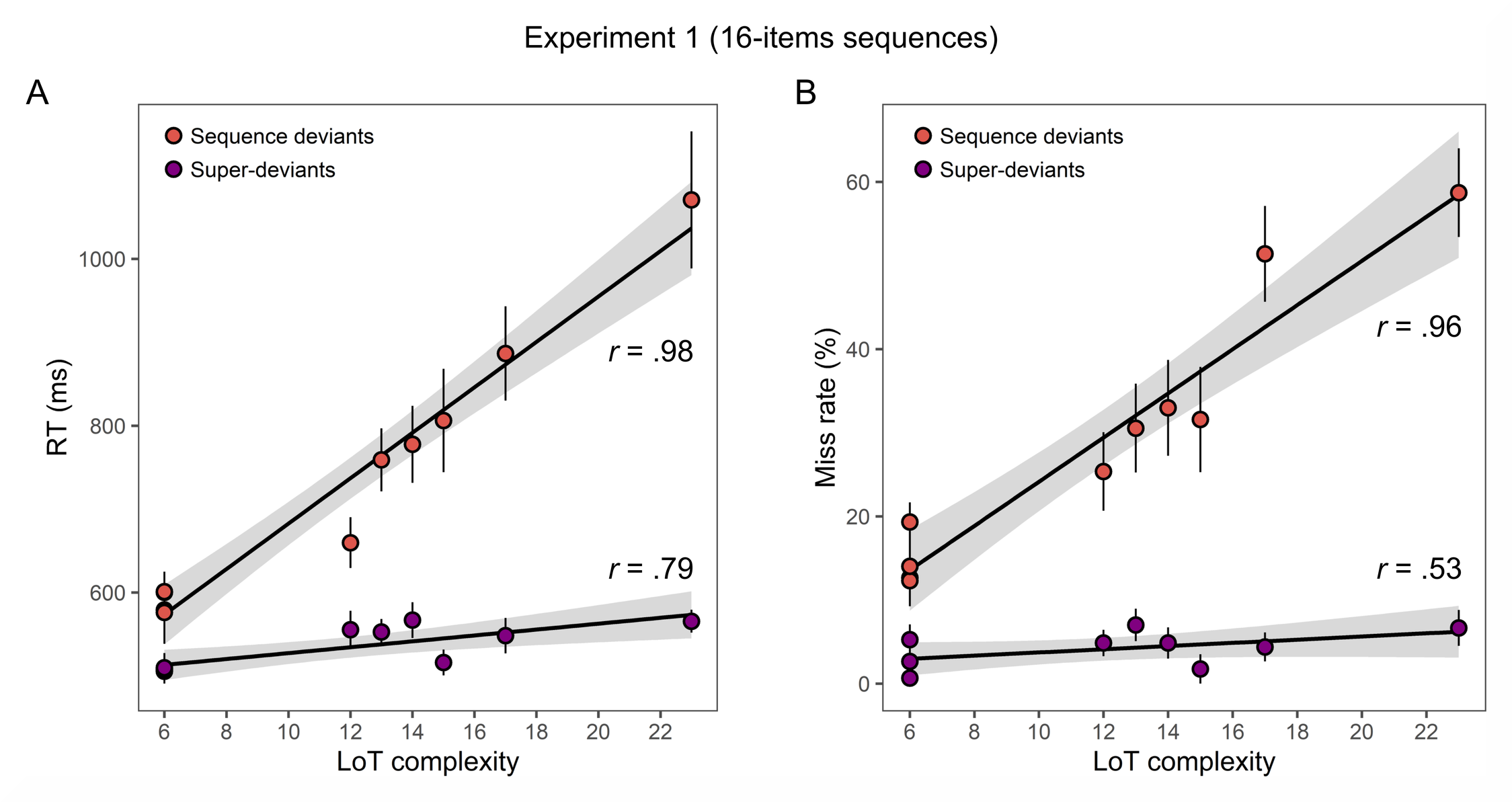

Supplement: S1 Fig — A) Average response time and B) average miss rate. (TIF) [file pcbi.1008598.s001.tif]

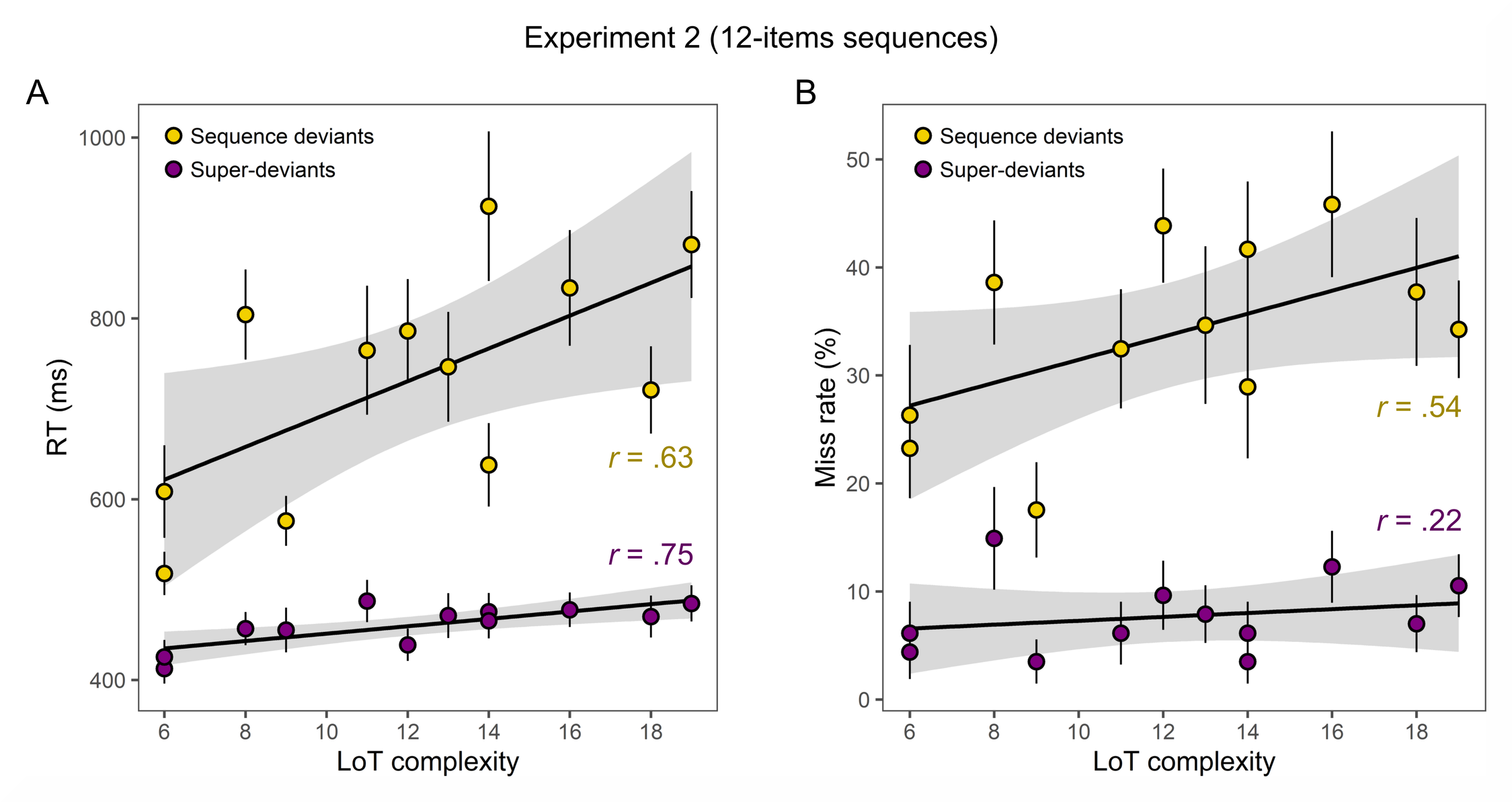

Supplement: S2 Fig — A) Average response time and B) average miss rate. (TIF) [file pcbi.1008598.s002.tif]

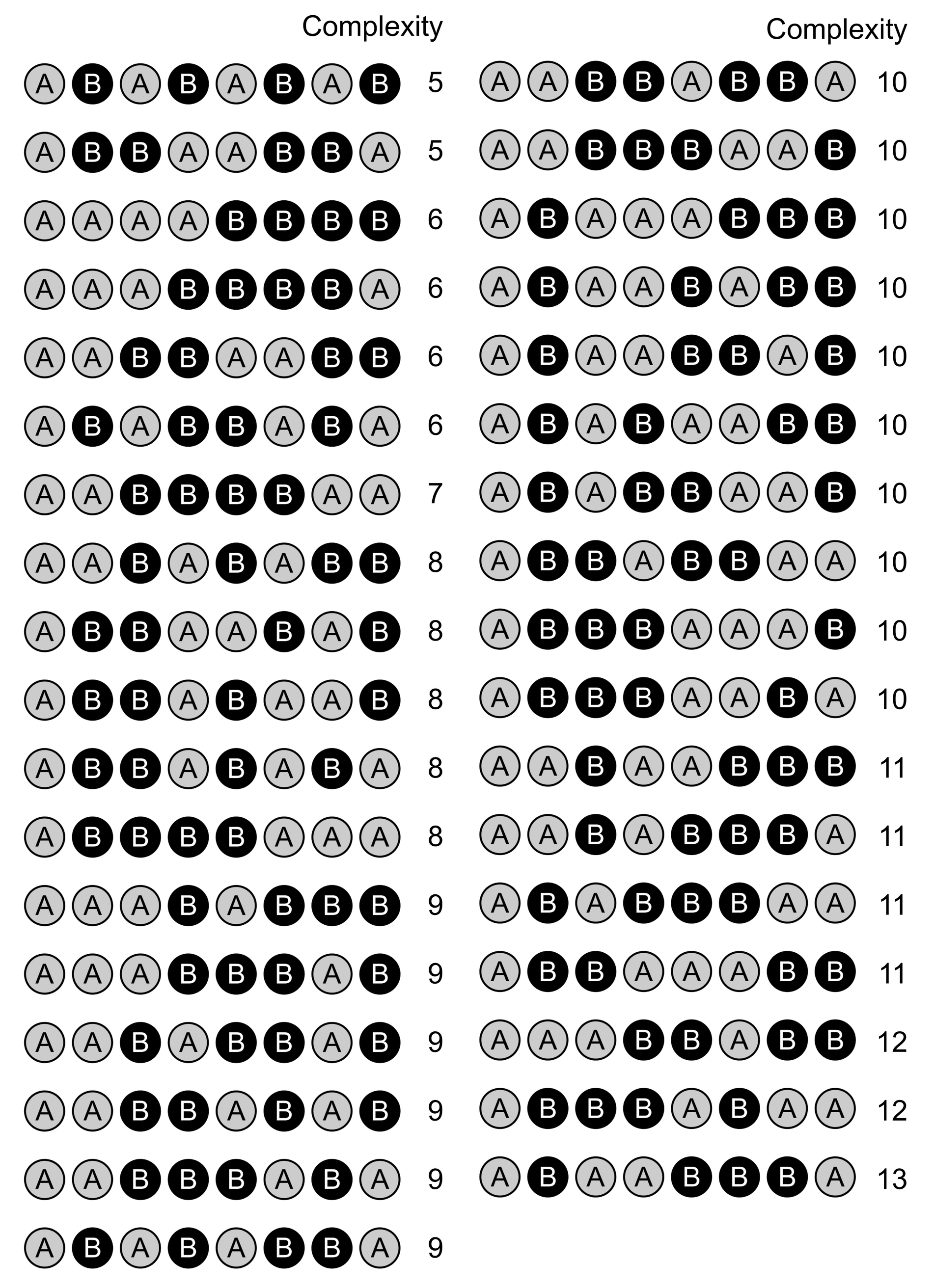

Supplement: S3 Fig — (TIF) [file pcbi.1008598.s003.tif]

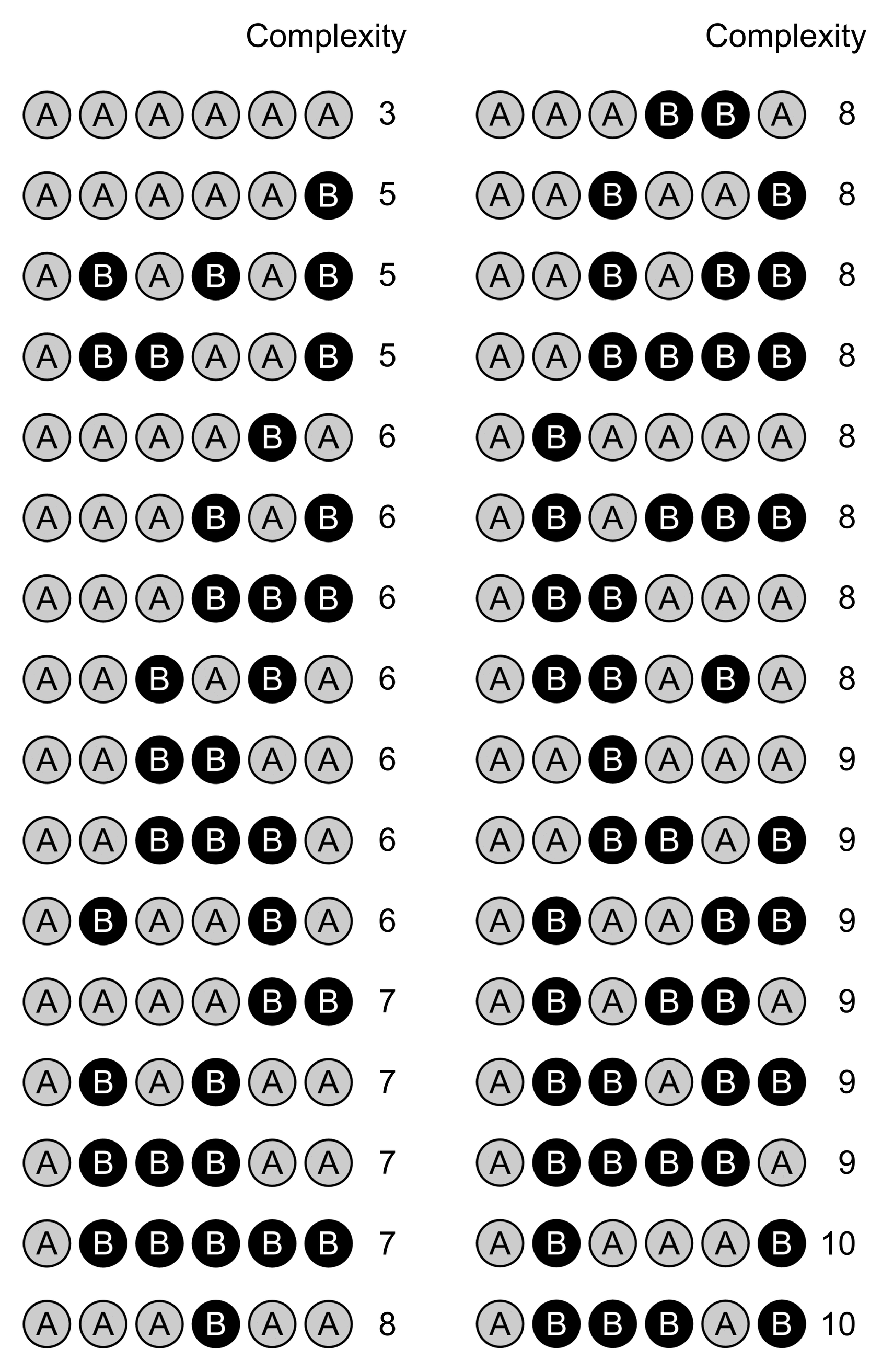

Supplement: S4 Fig — (TIF) [file pcbi.1008598.s004.tif]

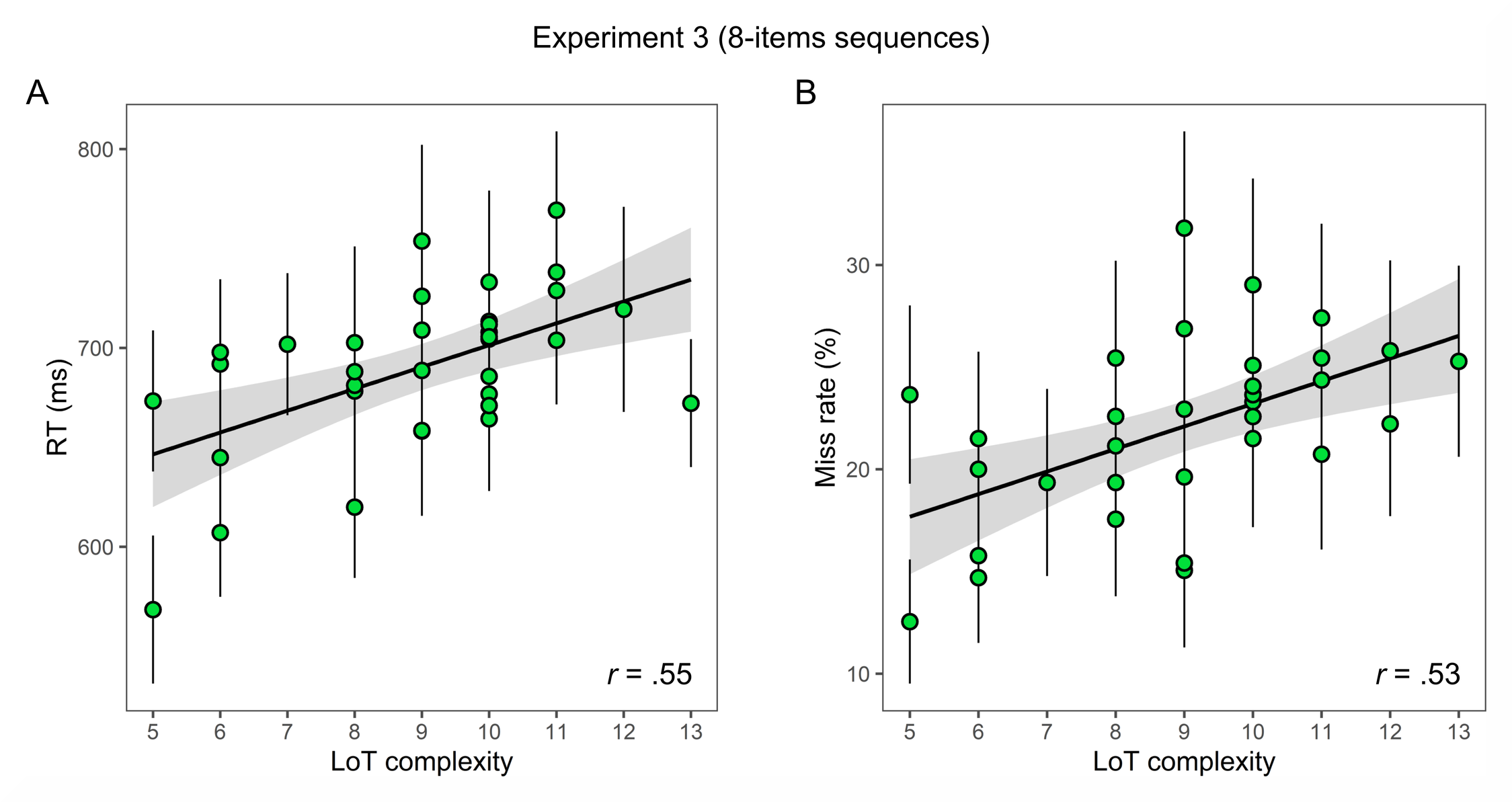

Supplement: S5 Fig — A) Average response time and B) average miss rate. (TIF) [file pcbi.1008598.s005.tif]

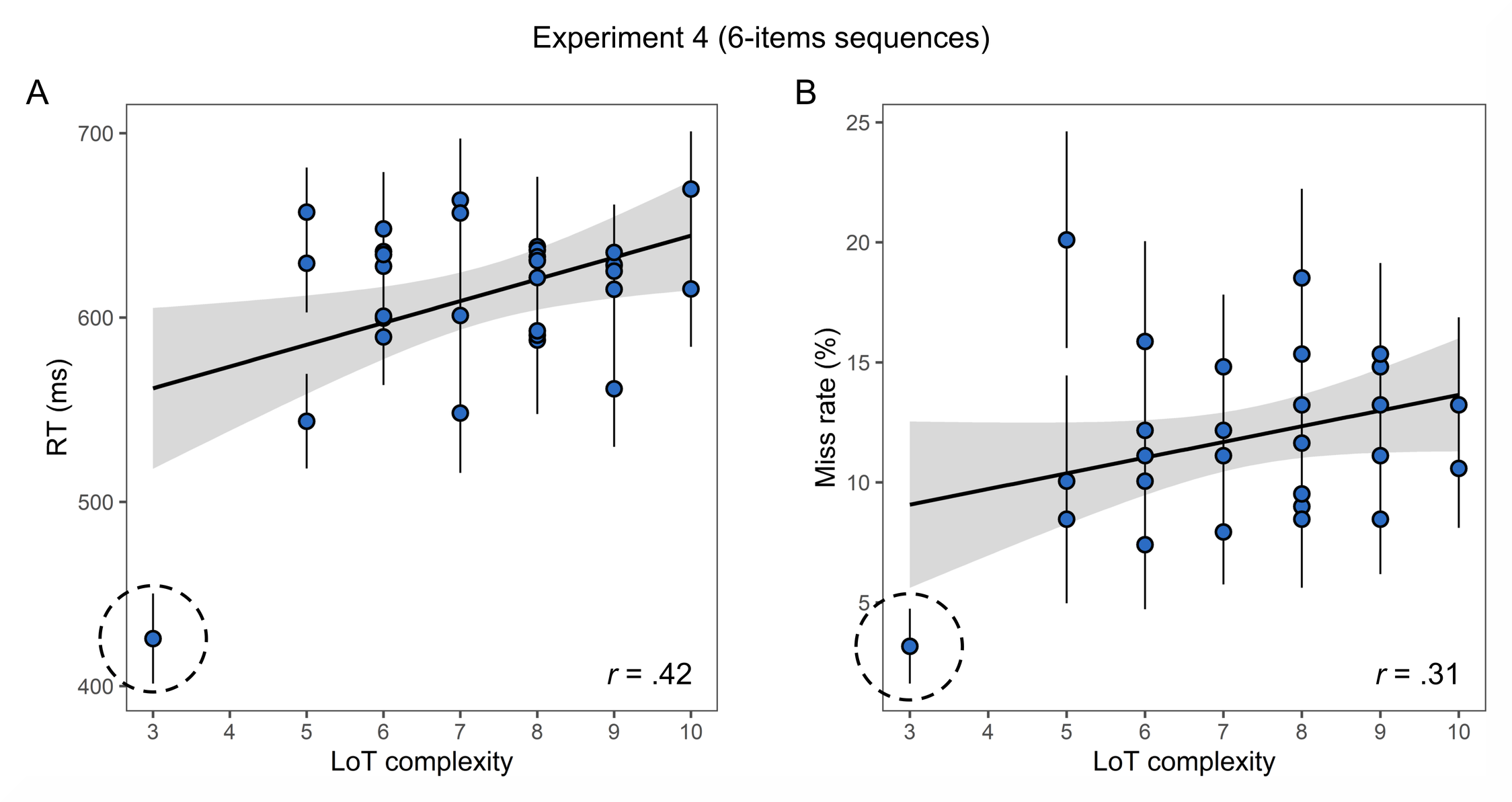

Supplement: S6 Fig — A) Average response time and B) average miss rate. Note: the circled dot highlights the performance for the sequence AAAAAA, which was excluded from some analyses. (TIF) [file pcbi.1008598.s006.tif]

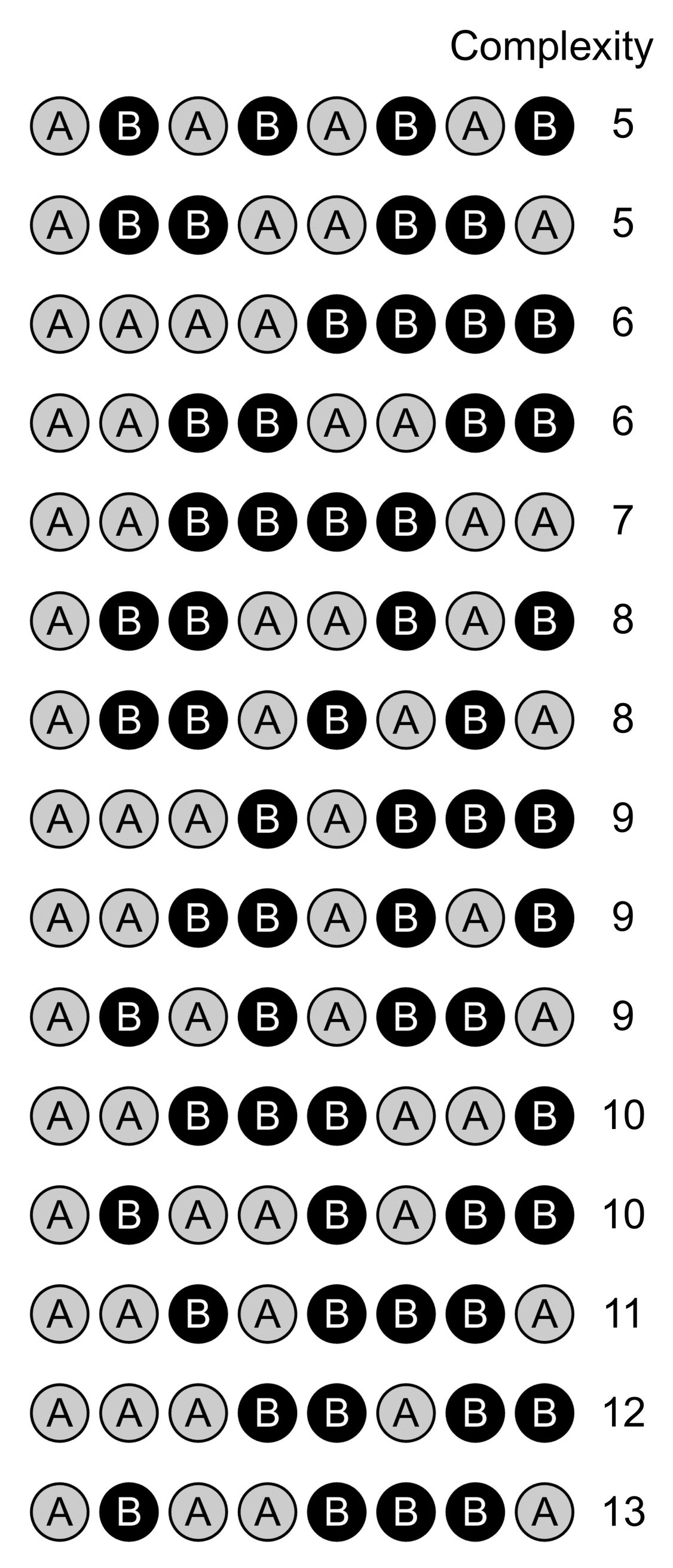

Supplement: S7 Fig — (TIF) [file pcbi.1008598.s007.tif]

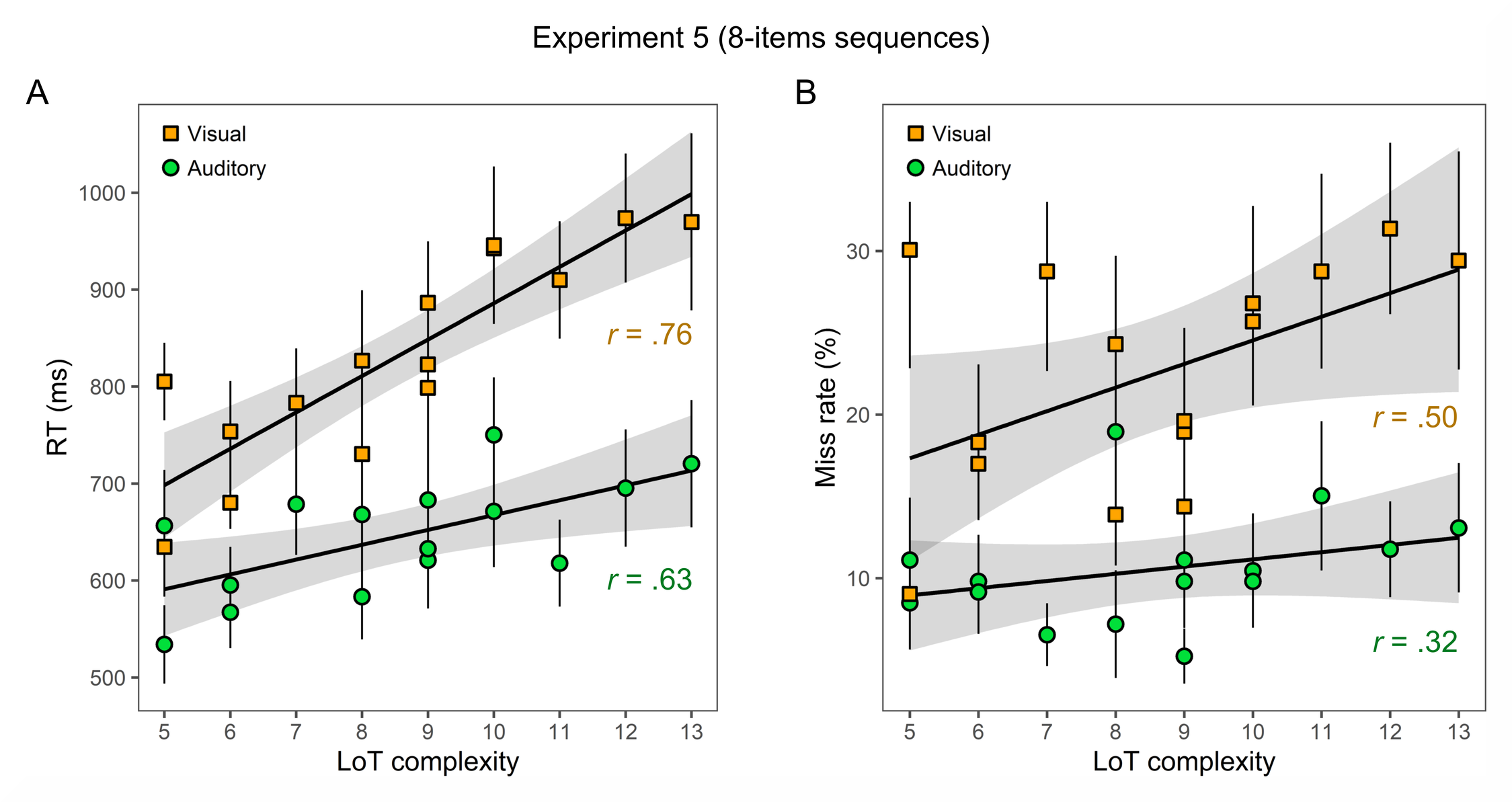

Supplement: S8 Fig — A) Average response time and B) average miss rate. (TIF) [file pcbi.1008598.s008.tif]

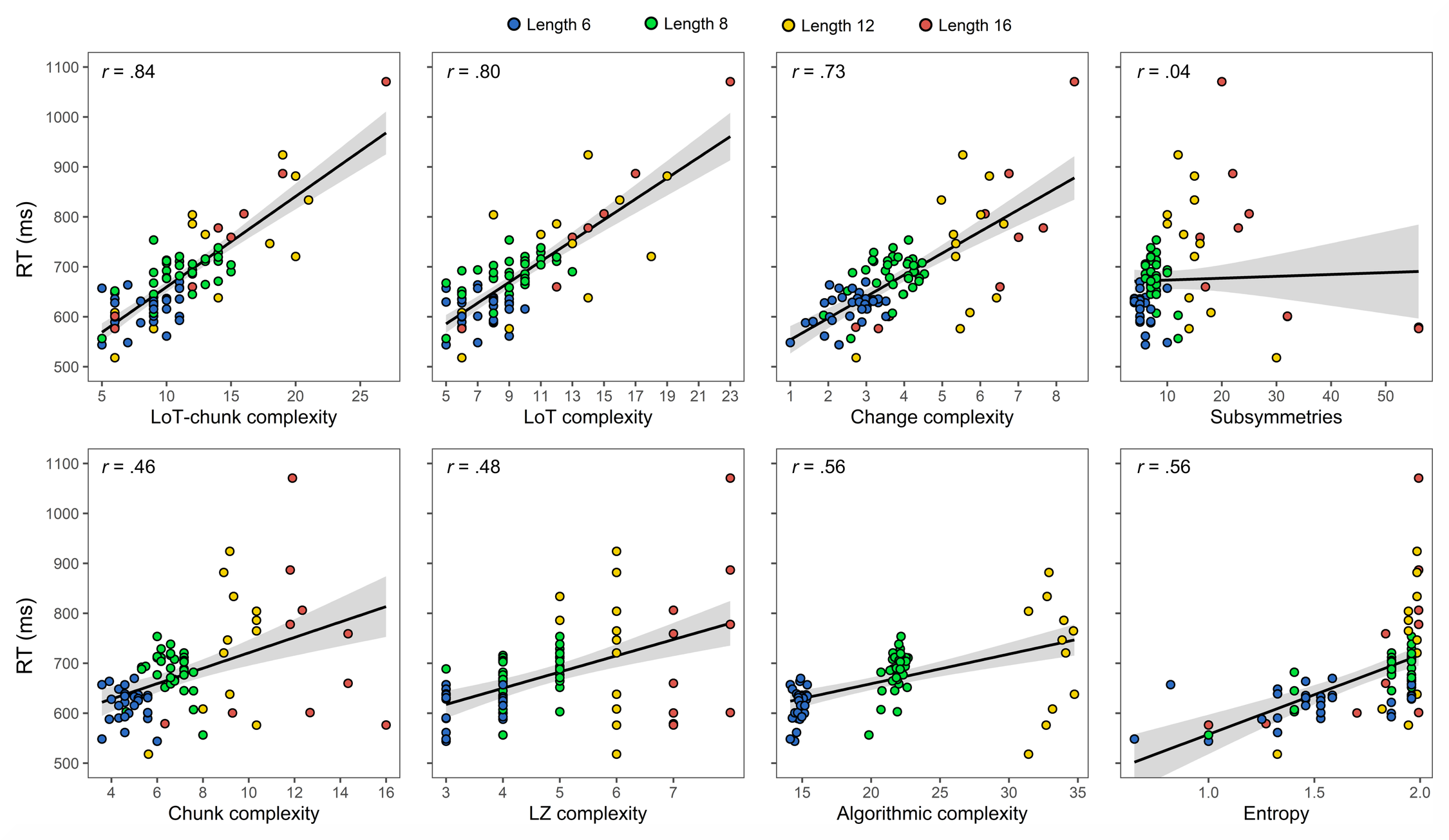

Supplement: S9 Fig — Note: 16-items long sequences (as well as one 12-items sequence) could not be included in the regression with algorithmic complexity. (TIF) [file pcbi.1008598.s009.tif]

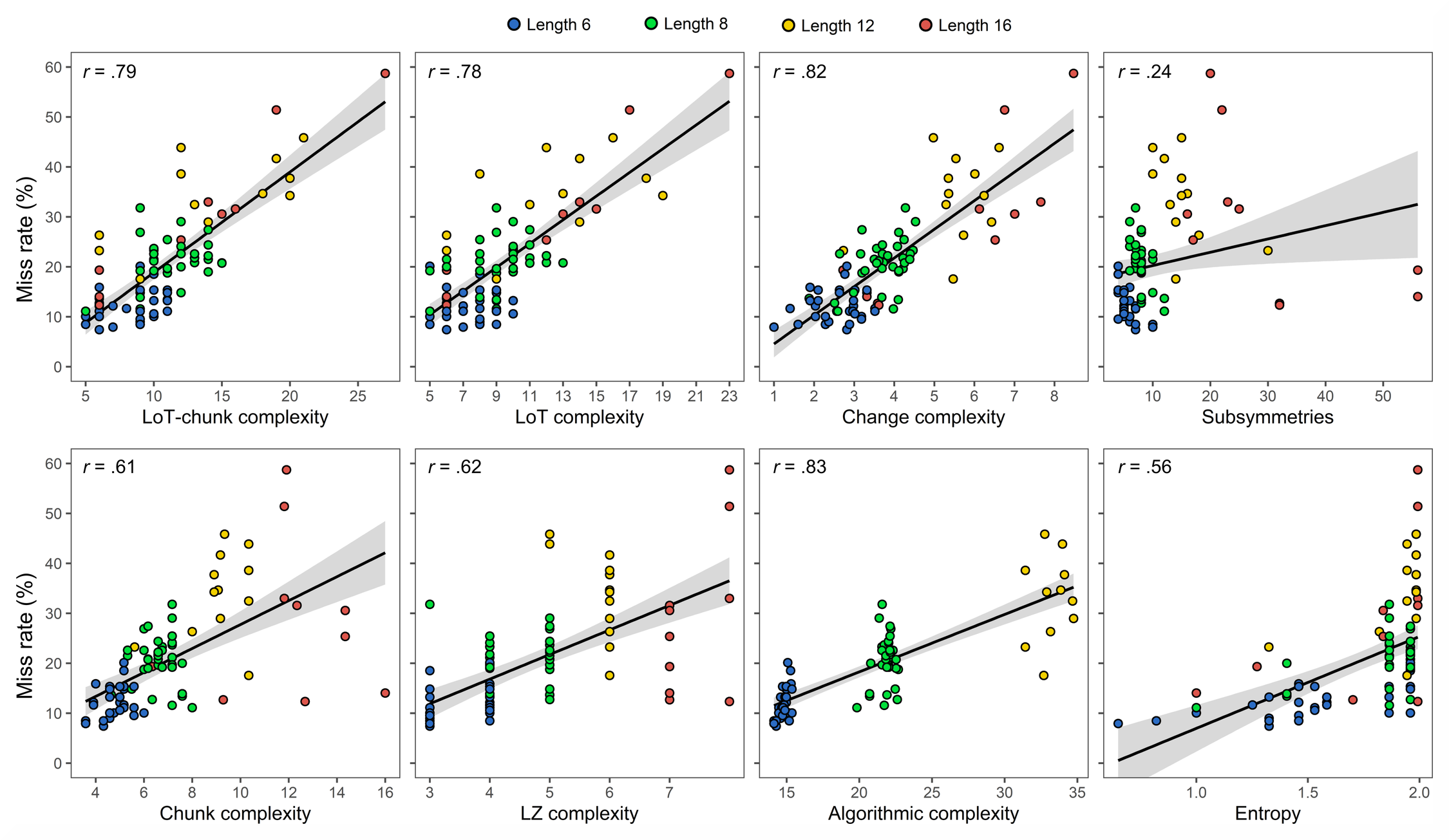

Supplement: S10 Fig — (TIF) [file pcbi.1008598.s010.tif]

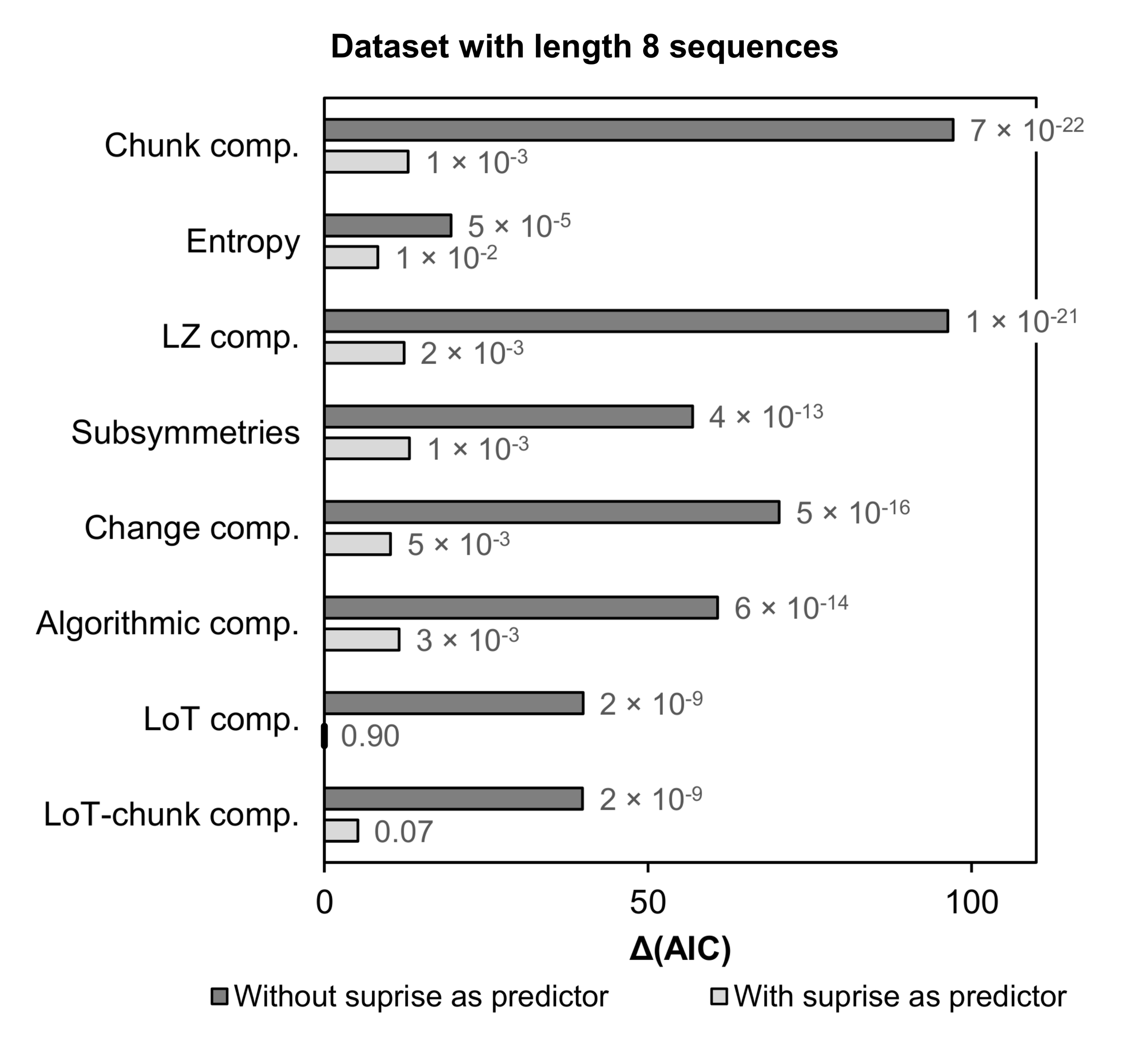

Supplement: S11 Fig — Δ(AIC) for the sixteen mixed models tested using a dataset including the task performance (LISAS) for sequences of length 8 (35 sequences).The fixed effect of interest is indicated along the vertical axis (all models included participants as a random effect and could include surprise as a covariate—light gray bars). Akaike weight for each model is also reported. (TIF) [file pcbi.1008598.s011.tif]
